# Temporally Robust Machine-Learning-Assisted Impedance Line-Shape Analysis for Quantitative QCM Biosensing with DNA Hybridization Validation

**DOI:** 10.3390/bios16100574

**Published:** 2026-10-12

**Authors:** Ceyhun E. Kırımlı, Elçim Elgün, Selin Yağmur Tuğtağ

**Affiliations:** 1 Department of Biomedical Engineering, Faculty of Engineering and Natural Sciences, Acibadem Mehmet Ali Aydinlar University, Istanbul 34752, Türkiye; 2 Department of Biomedical Engineering, Graduate School of Natural and Applied Sciences, Acibadem Mehmet Ali Aydinlar University, Istanbul 34752, Türkiye; eelgun@uwaterloo.ca (E.E.); selin.tugtag@live.acibadem.edu.tr (S.Y.T.); 3 Department of Computer Engineering, Faculty of Engineering and Natural Sciences, Acibadem Mehmet Ali Aydinlar University, Istanbul 34752, Türkiye

**Keywords:** quartz crystal microbalance, QCM biosensors, AI-assisted biosensing, impedance spectroscopy, resonance line-shape analysis, temporally blocked validation, interpretable machine learning, mRMR feature ranking, Kanazawa baseline, microfluidic concentration profiling, DNA hybridization, concentration prediction

## Abstract

Liquid-phase quartz crystal microbalance (QCM) biosensors are commonly quantified using scalar readouts such as frequency shift, dissipation, or bandwidth. Although useful, these descriptors discard resonance line-shape morphology that carries additional information for quantitative inference. Here, we present a machine-learning-assisted impedance line-shape workflow for temporally robust quantitative 10 MHz QCM biosensing. A passive microfluidic mixer generated controlled glycerol–water concentration gradients under constant total flow while complex impedance spectra were acquired in real time, and each sweep was parameterized using constrained Gaussian/Lorentzian models to obtain 52 physically interpretable descriptors spanning resistance, reactance, impedance magnitude, admittance components, and phase. The pipeline combines consensus outlier handling, training-fold mRMR feature ranking, and regression models evaluated under both shuffled five-fold cross-validation and a stricter temporally blocked protocol in which contiguous portions of the experiment are held out. The workflow achieved $R^{2}=0.948$ under shuffled validation and $R^{2}=0.823$ under seven-block temporal validation, the latter reducing prediction error by 48.4% relative to a row-matched Kanazawa frequency-shift baseline evaluated on the same sweeps. Feature-set ablation indicated that phase and conductance line-shape descriptors carry particularly strong temporally generalizable information. Applied to an experimental DNA hybridization assay in 1× PBS, the workflow reduced mean absolute percentage concentration error from 21.02% to 10.73%, a 48.9% improvement over conventional Δf calibration. Retaining resonance morphology therefore improves quantitative QCM biosensing beyond scalar frequency-shift calibration and provides an interpretable basis for AI-assisted electronic biosensors.

## 1. Introduction

Quartz crystal microbalance (QCM) sensing offers real-time label-free transduction and has become a workhorse for probing interfacial processes, adsorption, and viscoelastic films in liquids. In the rigid thin-film limit, the resonance frequency shift Δf follows Sauerbrey’s gravimetric relation [1]. In liquid-phase operation and for soft or viscoelastic loads, the response reflects coupled inertial, viscous, and elastic contributions; consequently, interpretation relies on hydrodynamic and viscoelastic frameworks such as the Kanazawa–Gordon bulk-loading model [2,3] and layered viscoelastic treatments (e.g., Voinova-type approaches) [4,5], with broader conceptual discussion by Johannsmann [6]. The modern practice and application breadth are summarized in recent QCM/QCM-D and acoustic-wave sensing reviews [7,8,9,10,11,12,13,14].

Each of these frameworks, however, rests on assumptions that liquid-phase biosensing routinely violates, and the resulting discrepancies are systematic rather than incidental. The Sauerbrey relation presumes a rigid, uniformly distributed, and acoustically thin film. Hydrated biomolecular layers satisfy none of these conditions: they dissipate energy and do not couple rigidly to the resonator, so the gravimetric inversion underestimates the acoustically coupled mass while the mass it does report exceeds the dry biomolecular mass because hydrodynamically entrained solvent is sensed as part of the layer [5]. Neither discrepancy is diagnosable from Δf alone. The uniformity assumption is likewise an idealization: the acoustic sensitivity is weighted toward the electrode center, so nominally identical loadings distributed differently across the active area produce different responses [15]. The Kanazawa–Gordon treatment describes a semi-infinite Newtonian liquid contacting a bare surface and therefore attributes the entire response to bulk viscosity and density; where an interfacial layer coexists with a changing bulk, as in any flow-based assay, it offers no means of apportioning the two. Surface roughness contributes further, trapping liquid that is sensed as rigidly coupled mass and displacing the resonance independently of any analyte [16,17]. Layered viscoelastic treatments relax several of these restrictions but at the cost of a poorly conditioned inverse problem: the general formulation requires five independent parameters to describe film thickness together with frequency-dependent viscoelastic properties, and the precision with which these can be recovered is limited by measurement noise so that distinct parameter combinations may reproduce the same data to within experimental uncertainty [18]. These constraints are particularly limiting in recognition-based biosensing, such as nucleic acid hybridization, where the analyte induces precisely the sort of thin, hydrated, and viscoelastic layers that confound these traditional models.

The approach taken here enriches the measurement as follows. Rather than sampling two scalars at each of several widely separated resonances, it samples the morphology of a single resonance densely, parameterizing the conductance, susceptance, resistance, reactance, magnitude and phase envelopes over narrow windows centered on their respective spectral features. The distinction is not that one method acquires more data than the other—a dissipation-monitoring instrument also fits a resonance curve, or an equivalent ring-down transient, in order to report Δf and ΔΓ at all—but that the reduction to two scalars is performed before any inference begins and by a model whose assumptions are seldom verifiable in situ. This yields an underdetermined problem where the adequacy of the model is never tested against the residual it discards. Retaining the line-shape parameters defers that reduction and allows the mapping to the quantity of interest to be estimated from the retained structure. The limitations of model-dependent inversion are recognized within the field, and model-independent treatments of QCM data have been developed in response [19]; the present work extends that direction by replacing the assumed physical model with an empirically estimated mapping that is subject to sufficiently strict validation to establish that the mapping generalizes beyond the conditions under which it was fitted.

Two practical consequences follow. First, temporal cadence: narrow windows around a single resonance can be swept rapidly enough to resolve a concentration gradient evolving on the timescale of a flow-based assay, which is the regime addressed here. Second, dimensionality is not in itself an advantage. The descriptors extracted from a single resonance are strongly intercorrelated, and a substantial fraction contribute little independent information, so the effective dimensionality is considerably smaller than the nominal count; establishing which descriptors carry generalizable signal should therefore be a central part of the analysis rather than a preprocessing detail.

The difficulty compounds when several mechanisms—bulk viscosity/density changes, interfacial slip, viscoelastic relaxation, surface roughness, or mixed-mode loading—contribute simultaneously and their effects overlap [6,11,12,17] since the number of contributing processes then exceeds the number of retained observables.

A direct route to increased information utilization is impedance-resolved (or admittance-resolved) analysis, where the full resonance line shape is acquired and parameterized rather than reduced to two metrics. Prior work has shown that full admittance/impedance spectra contain richer structure than a single frequency shift, and multivariate analysis of spectral descriptors can separate different physical contributions in liquid-phase QCM measurements [20,21,22,23,24]. A direct comparison of the two approaches on the same sensor has shown impedance analysis to outperform frequency-shift readout for viscosity determination in whole blood [25], indicating that the advantage persists in a complex biological medium. In particular, relating the complete admittance spectrum of a loaded resonator to material and interfacial properties has been shown to enable quantitative inference that is inaccessible to scalar reductions alone [26]. In parallel, electrochemical impedance spectroscopy has seen growing adoption of machine-learning and multivariate approaches for automated and more robust inference in complex settings, spanning impedimetric biosensing and bioanalytical sensing as well as energy-storage prognostics under variable operating regimes [27,28,29,30,31]. These developments motivate transferring similar AI principles—robust preprocessing, redundancy control, and cross-validated model selection—to impedance-resolved QCM line-shape data.

The application of machine learning to QCM measurements is established, although its prevailing implementation differs structurally from the approach adopted here. The predominant configuration is the chemical sensor array, in which multiple crystals bearing distinct selective coatings are exposed concurrently and the resulting pattern of frequency shifts is subjected to classification by artificial neural networks, principal component analysis or discriminant analysis for analyte identification and quantification in the gas phase [32,33]. Such architectures increase the number of transducers while retaining a single scalar descriptor from each and are effective where chemical selectivity is attainable through coating design. They are not directly applicable to the quantitative determination of a continuously varying property of the contacting medium by a single transducer, which characterizes liquid-phase QCM biosensing.

A more limited body of work exploits multiple electrical observables derived from a single resonator. Instrumentation permitting simultaneous acquisition of the series resonance frequency and motional resistance has been developed precisely because the frequency shift alone cannot distinguish the measurement target from the properties of the surrounding medium [34]. The limitation is general rather than incidental: reducing the response to two resonance parameters introduces cross-sensitivities whenever more than two physical quantities vary simultaneously and information that is recoverable from portions of the spectrum outside the motional branch has been shown to extend beyond what those two parameters convey [35], a limitation that is shared by related resonant fluid sensors [36]. The extension of this principle to a dense parameterization of the complete resonance morphology, as distinct from two or three lumped quantities, has not been systematically examined, nor has the predictive validity of models trained on such descriptors been established outside the temporal interval over which they were fitted. The reported evaluations in this domain rely predominantly upon randomly partitioned cross-validation, which is optimistically biased for temporally correlated acquisitions [37]; the question of generalization therefore remains substantially unresolved.

Nucleic acid detection by QCM is an ideal stress test for this underdetermination and remains an actively developed platform, with recent implementations extending to portable instrumentation and detection directly from clinical extracts [38]. The prevailing configuration employs a thiolated single-stranded capture probe immobilized on the gold electrode, with backfilling by short alkanethiols such as mercaptohexanol to orient the probe layer and reduce steric and electrostatic hindrance [39]; alternative attachment chemistries, including biotin–avidin coupling, trade sensitivity against response time and hybridization efficiency [40]. The approach has been applied to genetic diagnostic targets in real samples rather than synthetic oligonucleotides alone [41], and miniaturized devices now achieve single-base mismatch discrimination [42].

While detection is label-free, continuous, and mechanically simpler than optical alternatives, quantification is severely hampered by the scalar reduction. The hybridized layer is thin, hydrated, and viscoelastic—violating Sauerbrey assumptions—so the sensed mass includes variable amounts of hydrodynamically entrained solvent rather than the nucleic acid alone. This is not a theoretical concern: direct comparison of surface coverage inferred from QCM frequency shifts against an independent fluorescence displacement measurement has shown the QCM-derived figure to be substantially overestimated by an amount that depends on the buffer and the structure of the immobilized film [43]. Probe density exerts a further non-monotonic influence on hybridization efficiency through electrostatic repulsion and steric crowding at high coverage [39], and probe conformation itself responds to the interfacial environment [44]. The frequency shift is additionally confounded by non-specific adsorption and variation in ionic strength, which displace the resonance in the same direction as the target-induced response. Because that response saturates according to a Langmuir-type isotherm, quantification is reliable only across the pseudo-linear interval, with accuracy degrading as the plateau is approached.

These limitations are not attributable to the transducer, which registers the full consequence of the interfacial change, but to the reduction of its response to a single scalar. They motivate the present examination of whether a parameterization of the complete resonance morphology, interpreted by an empirically estimated mapping, recovers quantitative information that the scalar reduction discards.

In this work, we develop an impedance-resolved QCM inference framework that exploits full resonance line-shape information rather than reducing measurements to a two-scalar Δf/ΔD readout. To demonstrate this, we first apply the framework to a bulk-liquid microfluidic experiment featuring a constant total flow rate with a time-varying concentration gradient. For this dataset, descriptor redundancy is managed using mutual-information-based minimum redundancy maximum relevance (mRMR) ranking with top-k ablations, while transient artifacts and occasional fit failures are mitigated through staged outlier screening and a soft consensus ensemble filter. To avoid overestimating performance in a time-ordered sinusoidal experiment, we evaluate the pipeline under both shuffled five-fold cross-validation and a stricter temporally blocked protocol in which contiguous portions of the experiment are held out. We also use a row-matched Kanazawa baseline computed from the |Z| trough frequency position so that the physics-based comparator and the learned line-shape model are assessed on the same post-QC sweeps. This framework builds on our group’s prior work on impedance-resolved QCM feature engineering and ML-centric analysis workflows [45,46,47,48,49]. Finally, we extend the analysis to an experimental DNA hybridization QCM assay to demonstrate that the workflow generalizes from controlled bulk-liquid loading to recognition-based biosensing. The remainder of the paper details the experimental/microfluidic setup and spectral parameterization, the feature processing and learning pipeline, the validation strategy, and the comparative results and discussion.

## 2. Materials and Methods

### 2.1. Fabrication of Flow Cell with Passive Mixer

#### 2.1.1. CAD/CAM and Machining

The flow cell comprises three layers of 3 mm cast acrylic (PMMA). Geometry and toolpaths were prepared in Autodesk Fusion 360 and milled on an in-house-built CNC. Mixer features were cut with a 0.5 mm flat end mill inside a 3D-printed water bath for chip removal and thermal control. Unless noted, the feed rate was 300 mm/min and spindle speed 8000 rpm. All the ports, through-holes, and the QCM seat were machined before bonding.

#### 2.1.2. Passive Mixer Geometry

The middle layer contains an array of circular baffles (pillars) to enhance transverse advection. Pillar diameter is 1.25 mm; the gap between adjacent pillars is ≈0.53 mm. The channel height is 0.50 mm. These dimensions balance hydraulic resistance against fabrication constraints and respect the end-mill diameter. No claim is made here for the mixing efficiency of the baffle array: as set out in Section 2.2, the glycerol concentration delivered at the crystal face is determined by the commanded flow program and the geometry of the cell and does not depend on the performance of the array.

#### 2.1.3. QCM Sealing

A 10 MHz plano-convex AT-cut QCM (openQCM) of 14 mm diameter is sealed between two identical elastomer O-rings (11.1 mm inner diameter; 1.6 mm cord thickness) seated in concentric grooves. The crystal bears circular gold electrodes of 5 mm and 8 mm diameter on opposite faces, giving an acoustically active overlap area of 19.6 mm^2^. The upper O-ring defines the wetted footprint, exposing 96.8 mm^2^ of the crystal face to the flow at a pool height of 0.20 mm above the crystal surface; this height is set by the O-ring seat and is distinct from the 0.50 mm milled channel height of the mixer layer quoted in Section 2.1.2. The crystal is clamped between the fused (top + middle) assembly and the bottom plate, with O-ring compression preventing bypass flow.

#### 2.1.4. Electrical Interfacing

A custom PCB carries two spring-loaded pogo pins that contact the QCM electrodes from below through clearance holes in the bottom plate and O-ring groove. The PCB mates to a Keysight E4990A via the 16047E fixture. Full three-term open/short/load compensation was performed at the 16047E fixture plane prior to the measurement campaign. The descriptors used in the analysis are also relative line-shape quantities, normalized per parameter against the first sweep as described in Section 2.3.2 so that static fixture contributions—pogo-pin contact resistance, trace inductance and cable capacitance—are common-mode and cancel.

#### 2.1.5. Thermal Bonding (Heat Fusing)

The top and middle acrylic layers were permanently joined by controlled thermal bonding. To prevent warping under load, a snug MDF jig was CNC-cut to house the acrylic pair; the jig was clamped between flat aluminum plates, and binder clips provided uniform clamping pressure (layout provided in the Supplemental Information Section S1). The stack was heated in a box oven with a 2 °C/min ramp to 115 °C, held 30 min, then cooled to room temperature under load, yielding a clear void-free bond. Because both bonded layers are transparent PMMA, the bond line was inspected optically across the full mixer footprint and showed no voids, trapped air or unbonded regions. Channel integrity was further confirmed functionally over the measurement campaign, as described in Section 2.2.

#### 2.1.6. Final Assembly

**Figure 1:**
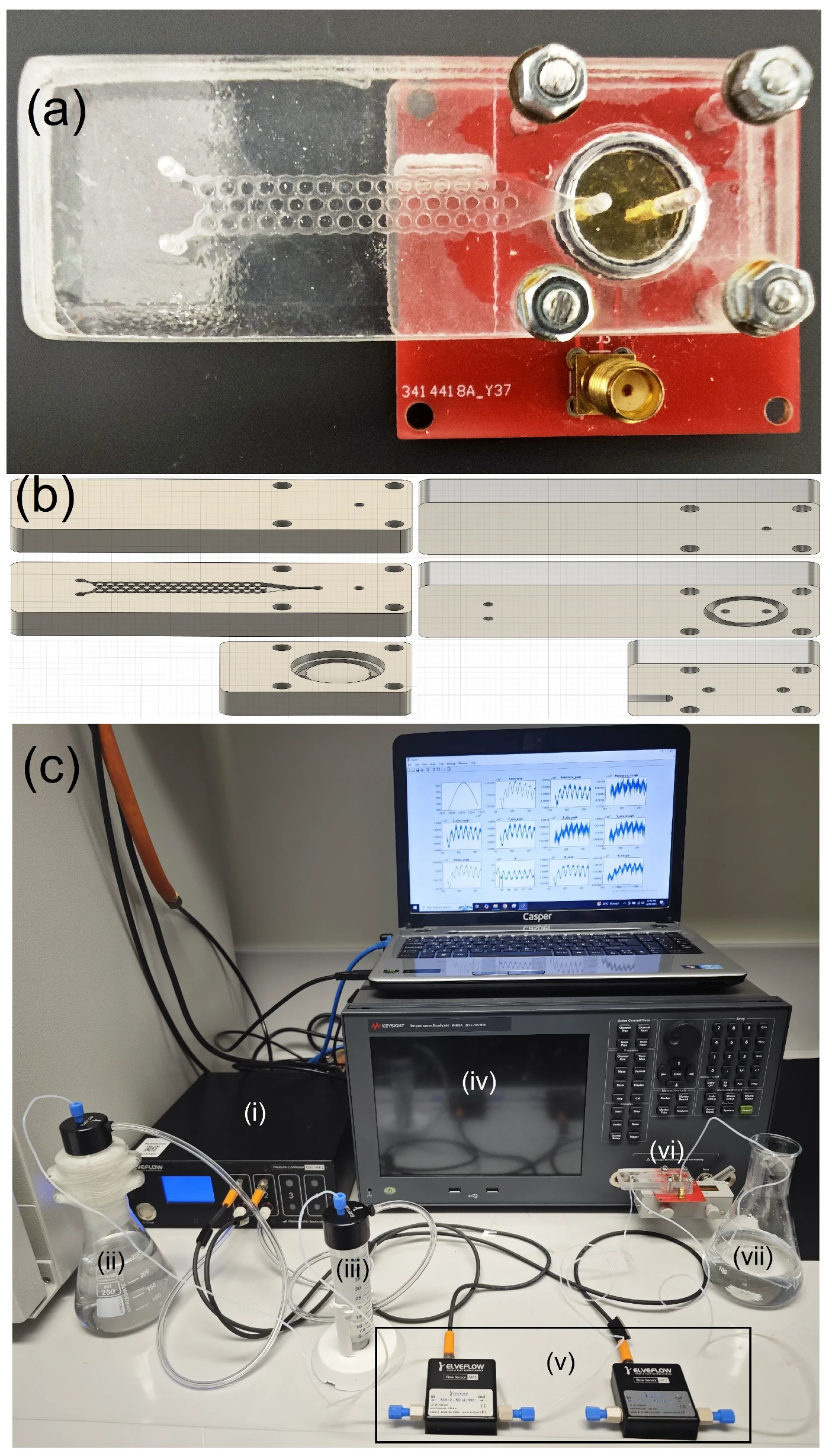
(**a**) QCM biosensor, flow cell and passive mixer. (**b**) Autodesk Fusion 360 drawings of machined faces of passive mixer. Left: top layers; right: bottom layers. (**c**) Flow setup and experimental components: (i) pressure controller, (ii) deionized water, (iii) glycerol solution, (iv) impedance analyzer, (v) flow sensors, (vi) flow cell with passive mixer, and (vii) waste container.

The bonded top–middle assembly, QCM, and bottom plate were stacked with the two O-rings and fastened using screws (Figure 1a). The PCB was then attached so the pogo pins engaged the electrodes. Figure 1a shows the assembled flow cell with the passive mixer; Figure 1b shows Autodesk Fusion 360 drawings of machined faces of the acrylic layers (top on the left; bottom on the right). The long pieces in Figure 1b correspond to the top and middle layers that were heat-fused. A step-by-step workflow, jig drawings, clamping pattern, and post-bond cleaning are provided in the Supplemental Information Section S1.

### 2.2. Flow Setup

Two inlet streams were driven by a dual-channel Elveflow OB1 MK3+ pressure controller equipped with inline flow sensors; each channel was regulated by proportional–integral (PI) feedback to enforce the programmed rates. Channel 1 (DI water) followed a sinusoidal profile between 30 and 50 μL/min with a 1 h period. Channel 2 (5% *v*/*v* glycerol) was commanded with an equal-amplitude sinusoid phase-shifted by 180°, varying between 0 and 20 μL/min. The phase offset maintained a constant total flow of 50 μL/min while modulating composition. Maintaining constant total flow is critical because QCM signals are sensitive to flow-induced hydrodynamic loading; fixing the flow rate reduces this source of variability. After merging, the baffle mixer produced a sinusoidal glycerol concentration at the sensor of 0–2% (*v*/*v*). The acquisition ran continuously for 7 h, comprising seven commanded concentration cycles at this 1 h period. Impedance sweeps were recorded throughout, yielding 681 sweeps at a mean interval of 37 s, of which 647 remained after the curve-fit quality control and consensus outlier removal described in Section 2.3.2 and Section 2.5; the retained record spans 6.92 of the seven commanded cycles. These seven cycles define the seven contiguous blocks used for the temporally blocked validation protocol of Section 2.8 so that each held-out block corresponds to one commanded concentration cycle. The record also provides a continuous functional test of channel integrity: across the 647 retained sweeps at a constant 50 μL/min there are no baseline steps, no spikes and no progressive decay of the modulation amplitude, which remains at 200 ± 20 Hz peak-to-peak across cycles. Bypass leakage would reduce this amplitude, delamination would drift the phase lag relative to the commanded program, and air ingress at a partial seal would introduce step discontinuities; none is observed. All the solutions, the flow cell, and the flow sensors were connected using PTFE tubing (inner diameter 1.0 mm; outer diameter 1.5 mm) with polyether ether ketone (PEEK) fittings and adapters to ensure chemical compatibility and low adsorption. For stable operation of the three-port passive mixer (two inlets, one outlet), the OB1 supplied positive pressure at the inlets; when the PI loop required negative gauge pressure to meet a setpoint, an auxiliary vacuum line (via the OB1 vacuum port/external source) was applied to the inlets. The outlet remained at atmospheric pressure and was routed to a waste container, which helped to prevent backflow and stabilized the junction while maintaining the programmed waveform. Flow-rate data sampled at 20 Hz were used to compute real-time concentrations and were interpolated to synchronize with the impedance-sweep timestamps. Figure 1c shows the flow setup and components. All the experiments were performed in a temperature-controlled laboratory nominally at 21 °C; the flow cell itself was not actively thermostatted. The thermal stability actually achieved was therefore bounded using the resonator as its own thermometer. After an initial settling transient spanning the first 93 sweeps, the baseline |Z| trough position drifted by −16.4 Hz over the remaining 554 sweeps, corresponding to less than 0.28 °C across the campaign. A thermal origin for the concentration modulation itself is excluded: the response remains phase-locked to the commanded pump program over all the cycles and the spectrum shows no discrete line at any non-harmonic frequency, whereas laboratory climate control has no coupling to the pump period. The settling transient has been retained rather than excluded from the analysis and is identified independently in the baseline position in the weakest held-out block of Section 3.5 and in the saturation of the Kanazawa inversion over the first sweeps.

### 2.3. Impedance Measurements and Curve Fittings

#### 2.3.1. Impedance Measurements

**Figure 2:**
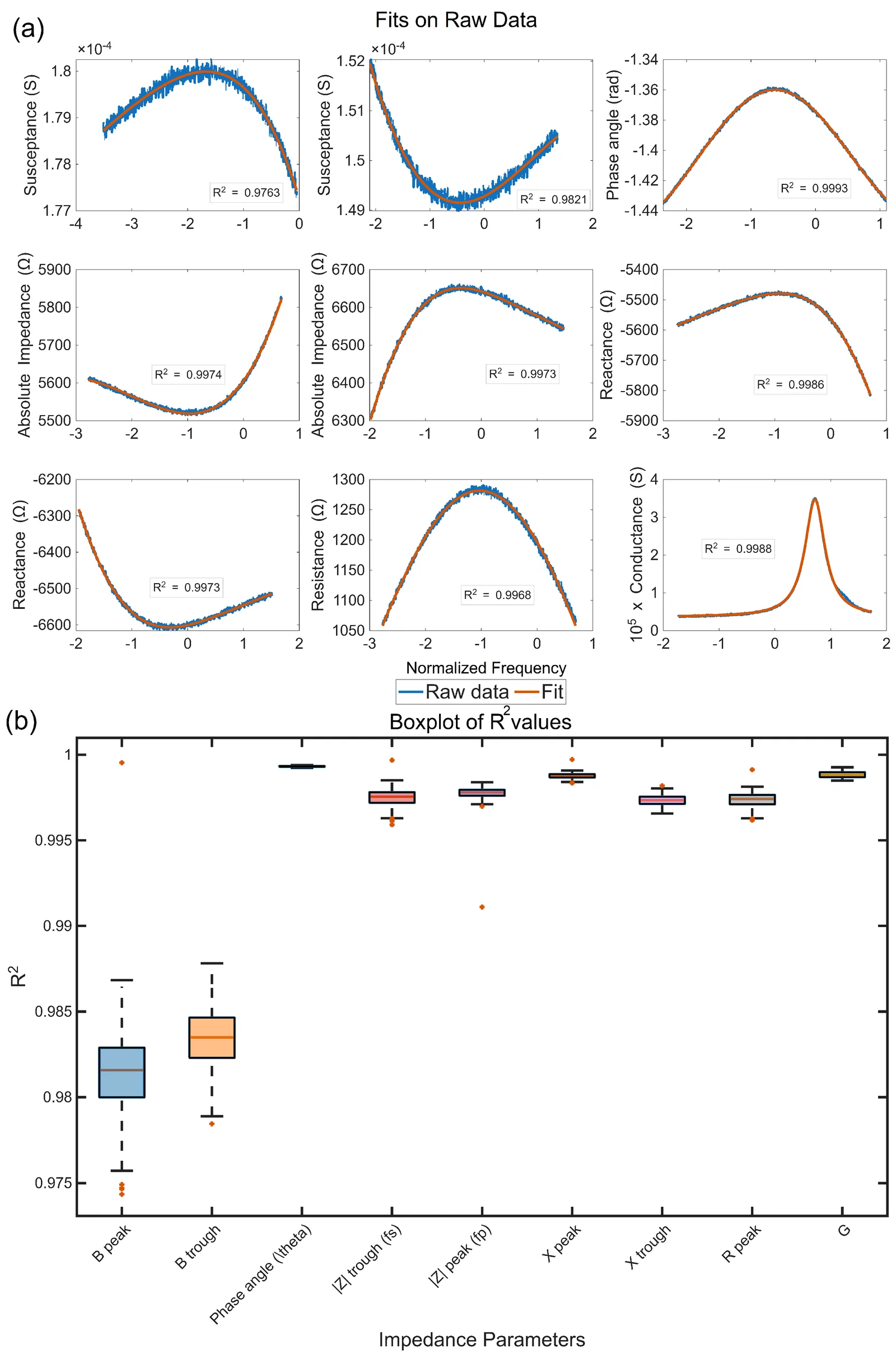
(**a**) Examples of impedance spectra around peaks and troughs. Blue represents raw data; orange represents fits. (**b**) Boxplots of $R^{2}$ values for curve fittings on experimental data giving impedance spectra of measurements.

Impedance spectra were collected around the fundamental resonance (∼10 MHz) with 1000 points per sweep and no on-instrument averaging. Each resonance feature was acquired in its own narrow moving frequency window, centered on the feature for all the measurements except conductance. The window sizes for the respective resonance features are listed in Table 1. Real-time feature tracking and recentering were applied to all the peaks/troughs except *G* using a peak-monitoring routine based on multiple-parabola resonance peak fitting [45]. The conductance peak was instead measured in a fixed 50 kHz window to guarantee that both tails of the line shape remained within the sweep even under slow drift. Fitting-window selection was predefined and feature-specific to preserve both central and wing information of each resonance observable; the complete windows are explicitly reported in Table 1 and representative wing coverage is visible in Figure 2a.

#### 2.3.2. Curve Fittings

Raw impedance data for each parameter was normalized for frequency only for each parameter separately by subtracting the first sweep’s mean and dividing by its standard deviation to retain shifts in frequency in time after fitting. The normalized spectral data were fitted with a two-term Gaussian model except for the conductance peak, which was fitted with a Lorentzian function with a constant offset. Equation (1) is given by

(1) $$L\left(x\right)=\frac{a}{{\left(x-b\right)}^{2}+c^{2}}+d$$

where *a*, *b*, *c*, and *d* are fitted constants. This parameterization yielded a total of 52 fit parameters. Importantly, the two-term Gaussian representation used for non-conductance features is a phenomenological decomposition of a single asymmetric resonance envelope (left-/right-wing-dominant components) and is not interpreted as two distinct physical resonances or modal coupling. The fit parameters of both the Gaussian and Lorentzian models are now defined explicitly, with annotated figures, in Supplementary Section S2. Fitting was done using lower and upper bounds, which were determined by unbounded fitting all 681 sweeps for each parameter and selecting 2nd and 98th percentiles of each fitted coefficient as lower and upper bounds to prevent overfitting (an unbounded fitting example may be found in Supplemental Information Section S2). Nine fits in $B_{peak}$ with an $R^{2}$ less than 0.95 were omitted from the dataset. A sample of fitted curves and raw impedance spectra is shown in Figure 2a, and a boxplot of all the $R^{2}$ values is plotted in Figure 2b.

### 2.4. Exploratory Data Analysis and Regression Models

#### Dataset

In constructing the dataset, each non-conductance resonance feature was fitted with a two-term Gaussian model, and a heuristic ordering was applied to the fitted components to ensure consistency across spectra. Specifically, the component with the larger fitted amplitude was designated as Gaussian 1 and its parameters were recorded with the suffix “1,” while the remaining component was designated as Gaussian 2 with the suffix “2.” The parameters from these two Gaussians, collected over all non-conductance features, yielded 48 input variables in total. The conductance peak was fitted separately with a Lorentzian function, and its four parameters were appended to the feature vector, bringing the total to 52 input parameters per sweep of each spectrum. The supervised target (output) associated with each 52-parameter vector was the corresponding glycerol concentration at the time of acquisition.

### 2.5. Outlier Removal

Outlier detection aims to identify and eliminate anomalous data points that deviate substantially from the general structure of a dataset. In experimental measurements, such deviations may arise from random noise, instrumentation errors, or uncontrolled environmental factors. However, distinguishing between genuine noise and boundary-region behavior is nontrivial as excessive filtering may lead to the loss of informative edge cases that represent physically meaningful system limits. Therefore, a balance must be maintained between preserving boundary behaviors and removing spurious noise that could distort downstream statistical or machine-learning analyses.

To establish this balance, the elbow method was employed to determine threshold values for each outlier detection algorithm. By plotting the number of flagged instances against a percentile or sensitivity parameter, the inflection (“elbow”) point reveals a natural transition between stable and unstable classifications. This approach provides an objective and reproducible threshold selection criterion, minimizing arbitrary parameter tuning.

Parameter bounding and omission of low-quality fits ($R^{2}<0.95$) effectively serve as a first-level outlier removal step, ensuring that only statistically reliable curve-fitting results are included in subsequent analyses.

In this study, outliers were handled in two stages. First, curve-fitting quality control (bounded parameterization and omission of low-quality fits, $R^{2}<0.95$) ensured that only statistically reliable descriptors entered downstream analysis. Second, multivariate anomaly scores were computed using three complementary detectors (local distance-based outlier factor, isolation forest, and Mahalanobis distance with shrinkage covariance). Scores were normalized and combined via a soft consensus score, and samples exceeding a robust threshold were excluded. Full definitions, hyperparameters, and threshold selection procedures are provided in the Supporting Information (Section S5).

### 2.6. Heatmap of Correlations

A correlation heatmap was constructed to evaluate pairwise linear relationships among all the fitted parameters. Correlation analysis quantifies the degree to which two variables vary together, with Pearson’s correlation coefficient ranging between −1 and +1, indicating the strength and direction of linear association. The heatmap provides a compact visual representation of these interdependencies, facilitating the identification of clusters of parameters that vary in a similar manner or exhibit inverse trends. This step serves both as an exploratory diagnostic to assess data coherence and as a guide for subsequent feature selection and dimensionality-reduction analyses.

### 2.7. Feature Importance Analysis

To determine the most informative parameters for predicting glycerol concentration, feature ranking was performed using mutual information (MI)-based minimum redundancy maximum relevance (mRMR) analysis. Feature importance quantifies the explanatory contribution of each variable to the target response, helping to identify those parameters that convey the most useful information while minimizing redundancy among correlated features.

The mutual information (MI) between a feature $X_{i}$ and the target variable *Y* measures the reduction in uncertainty about *Y* when $X_{i}$ is known. Unlike linear correlation coefficients, MI can capture both linear and nonlinear dependencies. It is formally defined as

$$I\left(X_{i},Y\right)=\int \int p\left(x_{i},y\right)log\frac{p(x_{i},y)}{p\left(x_{i}\right)p\left(y\right)}dx_{i}\;dy$$

where $p(x_{i},y)$ is the joint probability density function and $p(x_{i})$ and p(y) are the corresponding marginals. A higher MI value indicates a stronger statistical dependence between the feature and the target variable.

To ensure that the selected features are not only relevant but also non-redundant, the mRMR algorithm was applied. The mRMR criterion seeks to maximize the relevance of the selected feature subset S to the target variable while minimizing mutual information among features within the subset:

$$\max_{S}\left[\frac{1}{\left|S\right|}\sum_{x_{i}\in S}I\left(x_{i},Y\right)-\frac{1}{{\left|S\right|}^{2}}\sum_{x_{i},x_{j}\in S}I\left(x_{i},x_{j}\right)\right].$$

This formulation ensures that the resulting subset of parameters carries the maximum possible information about the response variable without duplication of information between features.

In this study, MI was employed as the primary dependency measure within the mRMR framework as it provides a deeper and more comprehensive assessment of feature relevance than linear correlation metrics. The resulting ranked list of features was subsequently used to evaluate regression model performance as a function of the number of included parameters.

### 2.8. Regression Modeling

Regression models were employed to quantitatively assess the effectiveness of the extracted and ranked features in predicting glycerol concentration. The objective was to evaluate the predictive utility of impedance line-shape descriptors and the effect of redundancy-aware feature ranking under a consistent cross-validation protocol rather than to claim a universally optimal model. To provide a balanced view of model interpretability and nonlinear learning capacity, three distinct classes of regression algorithms were considered.

#### 2.8.1. Linear Models

The lasso and elastic net regressions were implemented to evaluate the predictive power of sparse linear representations. Lasso regression imposes an $L_{1}$-norm penalty on the regression coefficients, forcing some weights to zero and thereby performing both regularization and feature selection. Elastic net combines the $L_{1}$- and $L_{2}$-norm penalties, providing a compromise between the sparsity of lasso and the stability of ridge regression. These models serve as interpretable baselines that reveal whether a linear combination of selected Gaussian- and Lorentzian-fit parameters can adequately describe concentration variations.

#### 2.8.2. Operator-Theoretic Model

The support vector regressor (SVR) was used as a kernel-based approach that projects the input space into a higher-dimensional feature space where linear regression is performed within a tolerance margin ε. SVR is derived from operator theory and optimization principles, where the regression function is constructed by minimizing structural risk rather than empirical error. This formulation allows the model to capture nonlinear trends while maintaining good generalization performance and resistance to overfitting.

#### 2.8.3. Algorithmic Ensemble Models

To further explore nonlinear relationships and variable interactions, ensemble-based tree models were trained, including random forest (RF) and gradient boosting regressor (GBR). Random forest aggregates predictions of multiple decorrelated decision trees to reduce variance and enhance robustness, whereas gradient boosting sequentially builds trees that correct the residual errors of the previous ensemble. These algorithmic models provide complementary insights into complex nonlinear dependencies that may not be captured by purely linear approaches.

Two validation protocols were used. First, a shuffled five-fold cross-validation analysis was performed to quantify within-distribution predictive capacity when training and validation folds are randomly interleaved across the full experiment. Second, a stricter temporally blocked validation was performed by sorting the data by acquisition order and dividing the post-QC dataset into seven contiguous held-out blocks. In each blocked fold, one temporal block was withheld for validation and the remaining blocks were used for training. This protocol tests whether the model can generalize to temporally separated portions of the experiment rather than relying on neighboring sweeps from the same concentration cycle.

All the analyses were performed on the same post-QC dataset (N = 647 sweeps) using fixed random seeds for reproducibility. Feature ranking (mRMR-MID) was recomputed within each training fold and then applied to the corresponding held-out fold to reduce information leakage. The models were evaluated using $R^{2}$, RMSE, and MAE on validation folds. For the physics-based comparator, a row-matched Kanazawa baseline was recomputed from the |Z| trough frequency position, treated as the series-resonance frequency $f_{s}$. The water reference was defined as the mean |Z| trough frequency of the 10 rows closest to zero flow-derived glycerol concentration; viscosity and density were computed at 21 °C and inverted using a lookup table constrained to the imposed 0–2%*v*/*v* concentration range.

### 2.9. Simulated Resonance Behavior Analysis Using the Butterworth–Van Dyke Equivalent Circuit

To support interpretation of the impedance line-shape descriptors extracted from experimental spectra, we performed an illustrative simulation using the Butterworth–Van Dyke (BvD) equivalent circuit. The BvD model provides a physically grounded representation of a quartz resonator through a motional branch $\left(R_{m},L_{m},C_{m}\right)$ in parallel with the static capacitance $\left(C_{0}\right)$. By imposing controlled perturbations in $R_{m}$ and $L_{m}$, the simulation generates corresponding physically consistent changes in the resonance response (e.g., resonance frequency, conductance, and phase), enabling a direct check of how the fitted Gaussian/Lorentzian descriptor set responds to known circuit-level variations. Importantly, this simulation is used to enhance the interpretability of the extracted features and to provide a controlled reference for trend analysis; it is not intended as a comprehensive surrogate for the full experimental loading physics.

Specifically, we simulated the experiment using the crystal parameters $R_{m}=5\;\Omega$, $L_{m}=9\times 10^{-3}\;H$, $C_{m}=28\times 10^{-15}\;F$, and $C_{0}=5\times 10^{-12}\;F$. A sinusoidal loading pattern was emulated by generating a 50Hz peak-to-peak shift in the phase-angle peak position over one period comprising 100 sweeps. This target shift was achieved by determining sweep-wise $L_{m}$ and $R_{m}$ updates through minimization of the peak-position error using the same real-time peak-tracking routine applied to the experimental data. The same window sizes and data resolution were maintained as in the experimental dataset. $R_{m}$ and $L_{m}$ were re-estimated after each round of nine sweeps, covering all troughs and peaks of the studied impedance parameters. The simulated spectra were then processed with the same downstream pipeline (feature extraction and filtering) up to the bivariate analysis stage, enabling like-for-like comparison of descriptor behavior between simulated perturbations and experimental observations.

### 2.10. Classical Kanazawa Model Based on Impedance

Kanazawa-based model predictions are calculated using half-bandwidth, Γ, which, for Newtonian fluids, shifts equally with $f_{r}$, fundamental resonance frequency [10]. The calculations involve peak position and baseline determination of the conductance and subsequent Γ detection, as explained in detail before [48]. The viscosity values calculated from the impedance data were then converted into data-oriented concentration, as explained before, and compared with the actual glycerol concentrations.

### 2.11. Experimental DNA Hybridization Assay and Concentration Prediction Analysis

To test whether the same impedance-resolved analysis framework can support a canonical recognition-based biosensing workflow, an experimental DNA hybridization assay was added using a 10 MHz QCM format. Prior to probe immobilization, QCM crystals were cleaned, electrically insulated, and functionalized with (3-Mercaptopropyl)trimethoxysilane (MPTMS) to generate a thiol-presenting surface. The thiolated surface was then reacted with sulfo-SMCC, a heterobifunctional linker containing a maleimide group for coupling to surface thiols and an NHS ester group for subsequent reaction with primary amines [45,50]. After linker activation and rinsing, a 5′-amine-modified capture probe containing a 12C PEG spacer between the terminal amine and the recognition sequence was immobilized on the surface. The capture-probe sequence was 5′-NH_2_–PEG_12_–CCTACGCCACAAGCTCCAAC-3′, and the immobilized probe was used to capture the fully complementary single-stranded DNA target, 5′-GTTGGAGCTTGTGGCGTAGG-3′. Both oligonucleotides were chemically synthesized and HPLC-purified by Eurofins Genomics. No human- or animal-derived material was used in this study. Unless otherwise stated, probe immobilization was performed by incubating the sulfo-SMCC-activated QCM surface with the amine-modified probe in PBS followed by rinsing and quenching of residual active ester groups. Target oligonucleotides were prepared by serial dilution in 1× phosphate-buffered saline (PBS) and introduced after a buffer-stabilization period. The time-course experiment was divided into a 0–30 min buffer/control interval followed by target-DNA injection at 30 min and post-spike monitoring until 60 min. The concentration series was analyzed both by conventional frequency-shift calibration and the impedance line-shape ML pipeline. For the conventional analysis, the final frequency shift was fitted with a Langmuir-type characteristic curve, and concentration uncertainty was estimated from replicate experiments performed within the linear region of the characteristic curve. Three replicate experiments were performed at each concentration. For each replicate, the frequency shift (Δf) was calculated as the average response between 57 and 60 min, after the signal had plateaued. These frequency shifts were then used to predict concentrations from the Langmuir fit. The same experimental data were used to train and evaluate the ML pipeline. The initial 30 min buffer/control interval of each run was labeled as zero concentration, and the same 3 min plateau interval was labeled with the corresponding target concentration for each DNA injection. The performance of both methods was quantified using the mean absolute percentage error (MAPE).

## 3. Results and Discussion

### 3.1. Curve Fitting and Parametric Representation

The impedance spectra around peaks and troughs are not symmetrical, as can be seen in Figure 2a. In order not to lose this vital information, functions retaining important parameters such as peak height, width and position were chosen to fit with high $R^{2}$ with the least amount of parameters. Asymmetry in Gaussians requires at least two terms. Fitting two-term Gaussians caused overfitting, as shown in the Supplemental Information, Section S2. Centers of individual Gaussians converge to outlier frequencies for a minimal increase in $R^{2}$, making a pattern with changing concentration difficult to emerge. To prevent this an unbounded fitting was used to determine boundaries for fit values, which lowered the $R^{2}$ values, as can be seen in Figure 2b, where the lowest $R^{2}$ value was 0.9743.

Each impedance spectrum was reduced to a compact physically meaningful set of line-shape descriptors. For all the parameters except conductance G, the spectral neighborhood is modeled with a two-term Gaussian mixture, capturing peak intensity, location, and width while remaining robust to minor asymmetries and baseline drift. The conductance peak, which is theoretically Lorentzian from the BvD model [11], was fitted with a Lorentzian function with constant offset to accommodate the experimentally observed baseline while preserving a compact four-parameter representation. Bounds on fit coefficients were derived from percentile statistics over all sweeps to prevent overfitting and ensure reproducibility. This procedure yields a 52-feature representation per sweep of each spectrum that preserves the core resonance information in a form that is suitable for multivariate analysis. We selected Gaussian and Lorentzian parameterizations, although third-order polynomial models also achieved high-$R^{2}$ fits to the line shape. This choice preserves interpretability by yielding direct descriptors of resonance morphology—peak position, peak amplitude, and width (FWHM-related quantities)—whereas polynomial coefficients are less physically transparent.

Figure 2b summarizes the goodness-of-fit across parameters: with the exception of susceptance, all the $R^{2}$ values exceed 0.995, indicating that the chosen parametric forms closely track the measured spectra. The slightly lower $R^{2}\left(\geq 0.97\right)$ for susceptance likely reflects its higher noise sensitivity and broader low-curvature region, but the residuals remain small and structureless relative to signal magnitude. Combined with the prior exclusion of low-fidelity fits and percentile-based coefficient bounds, these results confirm that the fitted features provide a faithful and stable surrogate of the raw spectra that is suitable for downstream correlation analysis, feature ranking, and regression.

### 3.2. Outlier Detection

#### 3.2.1. Ordering in the Venn Diagram

**Figure 3:**
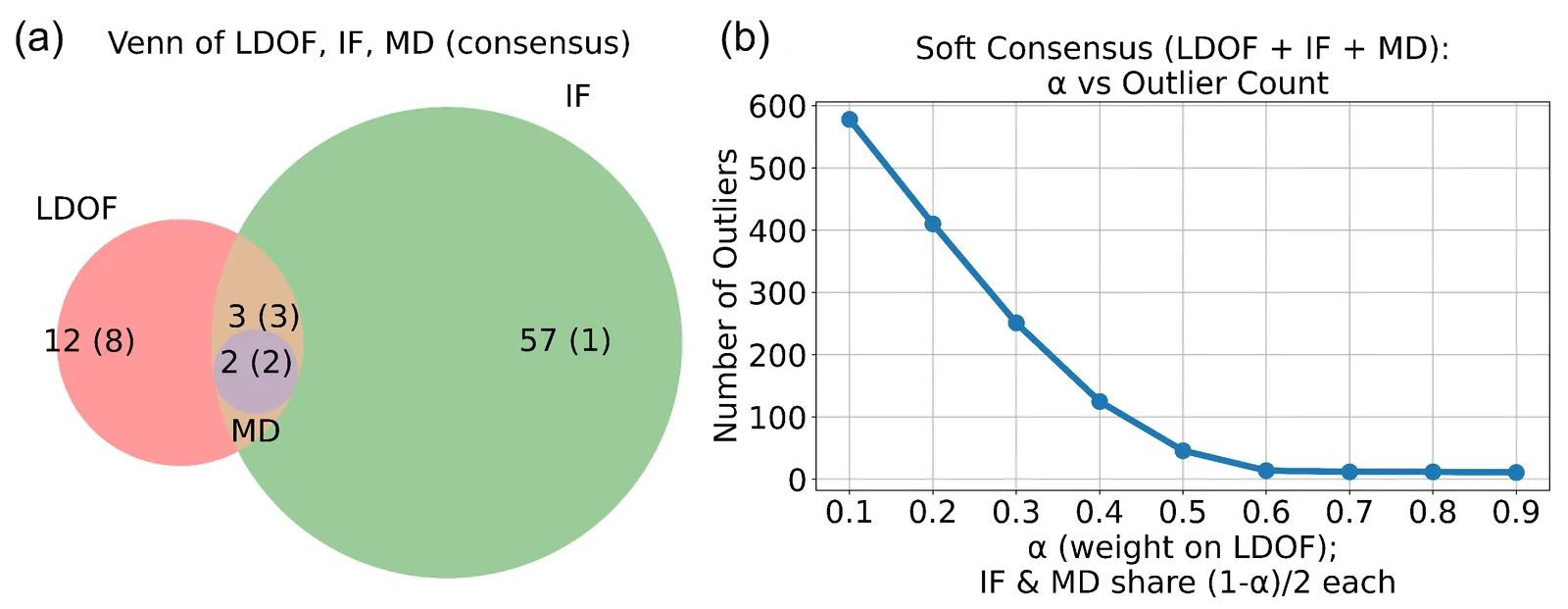
(**a**) Venn diagram of outlier points labeled independently by different (LDOF: local distance-based outlier factor; IF: isolation forest; MD: Mahalanobis distance) algorithms. First number is the number of data points labeled by each method. The number in parentheses denotes the number of those still labeled as outliers identified by consensus method. (**b**) Graph of number of outliers that the consensus method identified with respect to hyperparameter α.

Figure 3a shows that isolation forest (IF) removed the most points (62), local distance-based outlier factor (LDOF) fewer (17), and Mahalanobis distance (MD) the fewest (2), with MD being a subset of the other two. This ordering is consistent with each method’s sensitivity: IF isolates observations by random partitions and therefore readily flags sparse tails and small peripheral clusters (higher recall); LDOF emphasizes local density contrast and is stricter when a point lies near a cohesive neighborhood (moderate recall); MD assumes an approximately elliptical global structure with shrinkage covariance and typically marks only the most extreme directionally deviant points (highest precision; lowest count), hence the empirical pattern IF>LDOF>MD, and MD points tend to fall within the sets detected by IF/LDOF.

#### 3.2.2. Soft Consensus Method

None of the above three methods lead to an elbow graph, which is an indication that individual outlier detection methods do not catch boundary behavior (details may be found in Supplemental Information Section S5). To obtain a unified decision, a soft consensus ensemble method is employed that fuses continuous anomaly scores rather than binary votes. After robust normalization to [0,1] a weighted score is computed and an outlier is flagged when S≥τ (here τ=0.5 is the threshold). Because soft consensus aggregates sub-threshold but consistent evidence across detectors, it can identify points that do not appear in any single method’s binary set—hence additional outliers “outside” the Venn diagram. In contrast, hard consensus operates on binary masks only: a union rule (flag if any method flags) maximizes recall, while an intersection rule (flag only if all methods agree) maximizes precision but may miss borderline cases; neither leverages graded evidence.

#### 3.2.3. Elbow Selection of the Weight Alpha

The weights are parameterized as $w_{LDOF}=\alpha$ and $w_{IF}=W_{MD}=\left(1-\alpha \right)/2$. Plotting the number of flagged outliers versus α produces a characteristic elbow curve, seen in Figure 3b: large changes in count for small α (dominated by IF/MD), followed by a region of diminishing returns as α increases (greater emphasis on LDOF’s local-density view). α is selected at the elbow—where the slope drops sharply—because it balances noise removal against boundary preservation and yields stable downstream behavior. In our study, this criterion led to α=0.6.

### 3.3. Correlations

**Figure 4:**
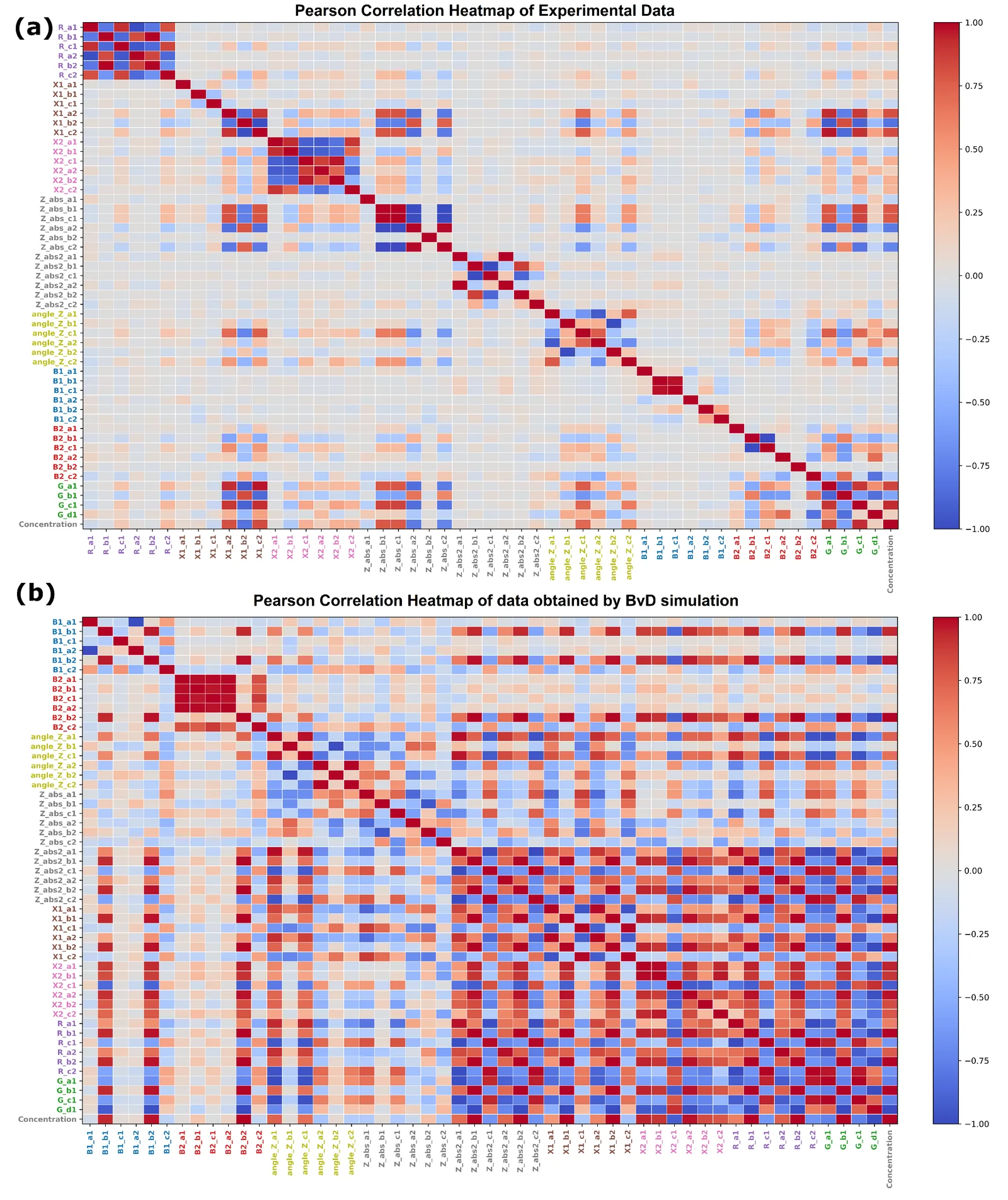
(**a**) Heatmap of Pearson correlation values computed for experimental data. (**b**) Heatmap of Pearson correlation values computed for BvD simulation results.

Figure 4 shows a heatmap of the correlation matrix where the matrix has a unity diagonal due to perfect self-correlation. When focused around the diagonal—for $R,X_{trough}$, both the Gaussian terms’ fit parameters and *G*—all the fit parameters are highly correlated. $\Theta_{Z},{\left|Z\right|}_{peak}$ and ${\left|Z\right|}_{trough}$ show moderate correlation, and, for $X_{peak}$, only the second Gaussian term shows correlation. $B_{peak}$ shows correlation mainly for the fitted center parameter *b* and the width-related parameter *c* for both Gaussian terms, and $B_{trough}$ shows the same behavior primarily for the first Gaussian term. The Gaussian *c* parameter was therefore treated as a width-related descriptor without assigning an independent mechanistic interpretation to individual Gaussian components. Peaks and troughs correspond to the series and parallel resonance frequencies of the Butterworth–Van Dyke equivalent circuit model (BvD).

$$f_{s}=\frac{1}{2\pi \sqrt{L_{m}C_{m}}}\;\mathrm{and}\;\;f_{p}=f_{s}^{lossless}\sqrt{\left(1+\frac{C_{m}}{C_{0}}\right)}$$

where $C_{m}\ll C_{0}$. However, BvD is a simplified model, and variations in the BvD model and computational studies on the BvD itself have been studied extensively in the literature to better represent the behavior of QCM under load from Newtonian fluids [11,24,51,52]. As both $f_{s}$ and $f_{p}$ shift to lower frequencies according to the BvD model upon changes in motional inductance ($L_{m}$) and motional resistance ($R_{m}$) due to bulk fluid immersion, more of the parameters were expected to be correlated. Although the asymmetrical impedance peaks were captured fully in simulation, as shown in Supplemental Information Section S3, a bivariate analysis of the simulation results of the BvD model shows more of the parameters to be correlated, and the pattern of those correlations from stronger to weaker is also different. These results suggest that there is a more complicated underlying mechanism in QCM’s response to changes in the viscosity of the Newtonian fluid that is not fully captured by the simplified BvD formulation used here, suggesting additional contributions beyond the modeled $R_{m}/L_{m}$ perturbations. Moreover, although in reality all the peak positions showed high correlations in terms of the concentration with sinusoidal patterns (the figures may be found in Supplemental Information Section S9), the fit parameters in this study captured more nonlinear behavior when combined with regression models and predicted the changes in concentration with significantly higher accuracy.

### 3.4. Parameter Importance

A linear dimensionality analysis of the dataset is applied using principal component analysis (PCA). The results may be found in Supplemental Information Section S6. Table 2 shows the importance of parameters in explaining the variation in concentration. Other statistical methods to determine parameter information may be found in Supplemental Information Section S4. The most important parameters are the height of the dissipation peak, followed by the second Gaussian term of the phase angle. The phase angle peak is situated in between the $f_{s}$ and $f_{p}$ peaks, and, as both frequencies shift, the phase angle peak shifts. The results indicate that the tail of the peak closer to $f_{s}$ is more responsive to concentration changes. The peak position of the conductance is the third parameter, followed by the reactance trough’s second Gaussian term’s width. In the top-10 most important parameters three of the four terms of the conductance peak are followed by three terms of the phase angle covering both of its tails, which is followed by two terms of |Z|’s $f_{s}$ trough and both tails’ amplitudes. It is important to note that the sorting algorithm employed here increases the convergence of the regression algorithm with as few parameters as possible by penalizing unsorted parameters with how redundant they are with respect to already selected parameters in explaining concentration. This means, if one has to pick a parameter out of this ranking, a parameter that is closer to the end may perform quite well alone. (The bar graphs of mRMR-MID scores may be found in Supplemental Information Section S7.)

### 3.5. Regression Models and Temporally Blocked Validation

**Figure 5:**
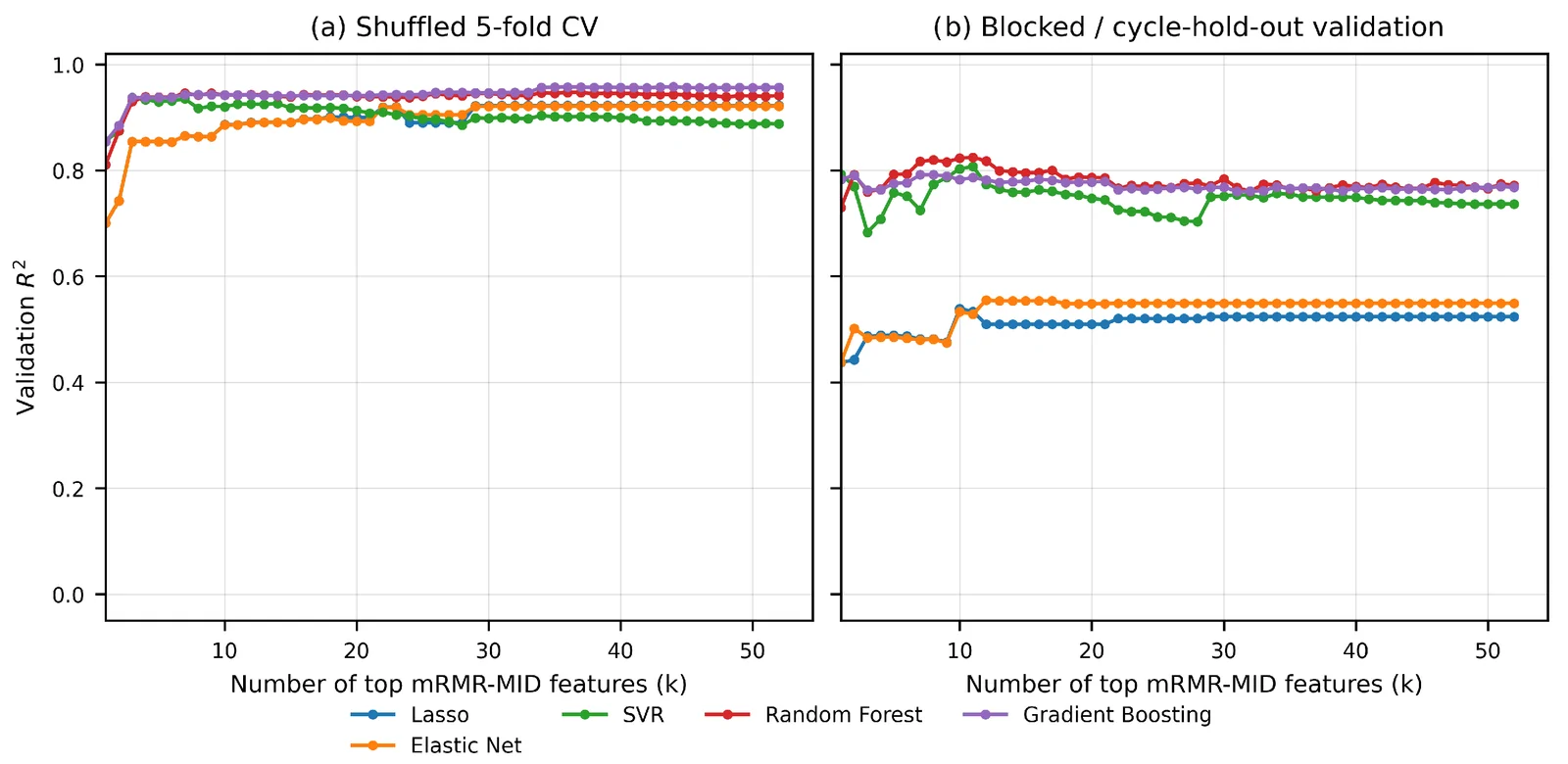
Model performance under shuffled and temporally blocked validation. (**a**) Shuffled five-fold cross-validation performance, plotted as validation $R^{2}$ versus the number of top-ranked mRMR-MID descriptors. (**b**) Matched blocked/cycle-hold-out validation using the same feature-ranking procedure and model families. The decrease from shuffled to blocked validation quantifies the loss in apparent performance when temporally adjacent sweeps are no longer shared between training and validation folds.

To evaluate the predictive value of the fitted line-shape parameters and to verify the benefits of feature selection, the lasso, elastic net, SVR, random forest, and gradient boosting regressors were trained using mRMR-MID-ranked descriptors. Performance was evaluated as a function of the top-*k* features under two validation protocols (Figure 5). In the shuffled five-fold setting, the validation folds contain randomly drawn samples across the full experimental sequence. This protocol measures within-distribution predictive capacity and confirms that the line-shape descriptors support high-accuracy concentration inference; the best shuffled reference model achieved $R^{2}=0.948$, RMSE=0.162%v/v, and MAE=0.128%v/v. The regression model results are given in detail for 5-fold CV in Supplemental Information Section S8. The best models’ prediction performance is shown in Supplemental Information Section S10.

Because the experiment follows a temporally ordered sinusoidal concentration program, a stricter blocked validation was added to test temporal generalization. In this protocol, seven contiguous held-out blocks were used so that, apart from the sweeps immediately adjacent to each block boundary, no neighboring sweeps from the held-out block were available during training. As expected, the performance decreased relative to shuffled cross-validation, but the line-shape descriptors retained substantial predictive value. Using the top-11 mRMR-MID descriptors under blocked out-of-fold validation, the random forest model achieved $R^{2}=0.823$, RMSE=0.298%v/v, and MAE=0.217%v/v; all the blocked metrics are computed on the pooled out-of-fold predictions across the seven held-out blocks. The feature count was selected at the optimum of the blocked validation curve over the full range k=1 to 52. Performance rises steeply from $R^{2}=0.726$ with a single descriptor, plateaus at $R^{2}=0.815$–0.823 across k=8–12, and then declines to $R^{2}=0.769$ when all 52 descriptors are used. Restricting the model to approximately ten line-shape descriptors is therefore not merely economical but measurably better than using the full set, consistent with the redundancy that mRMR-MID is designed to remove. This result is more conservative than the shuffled five-fold estimate and therefore provides a stronger basis for claiming robustness in time-ordered QCM measurements.

**Figure 6:**
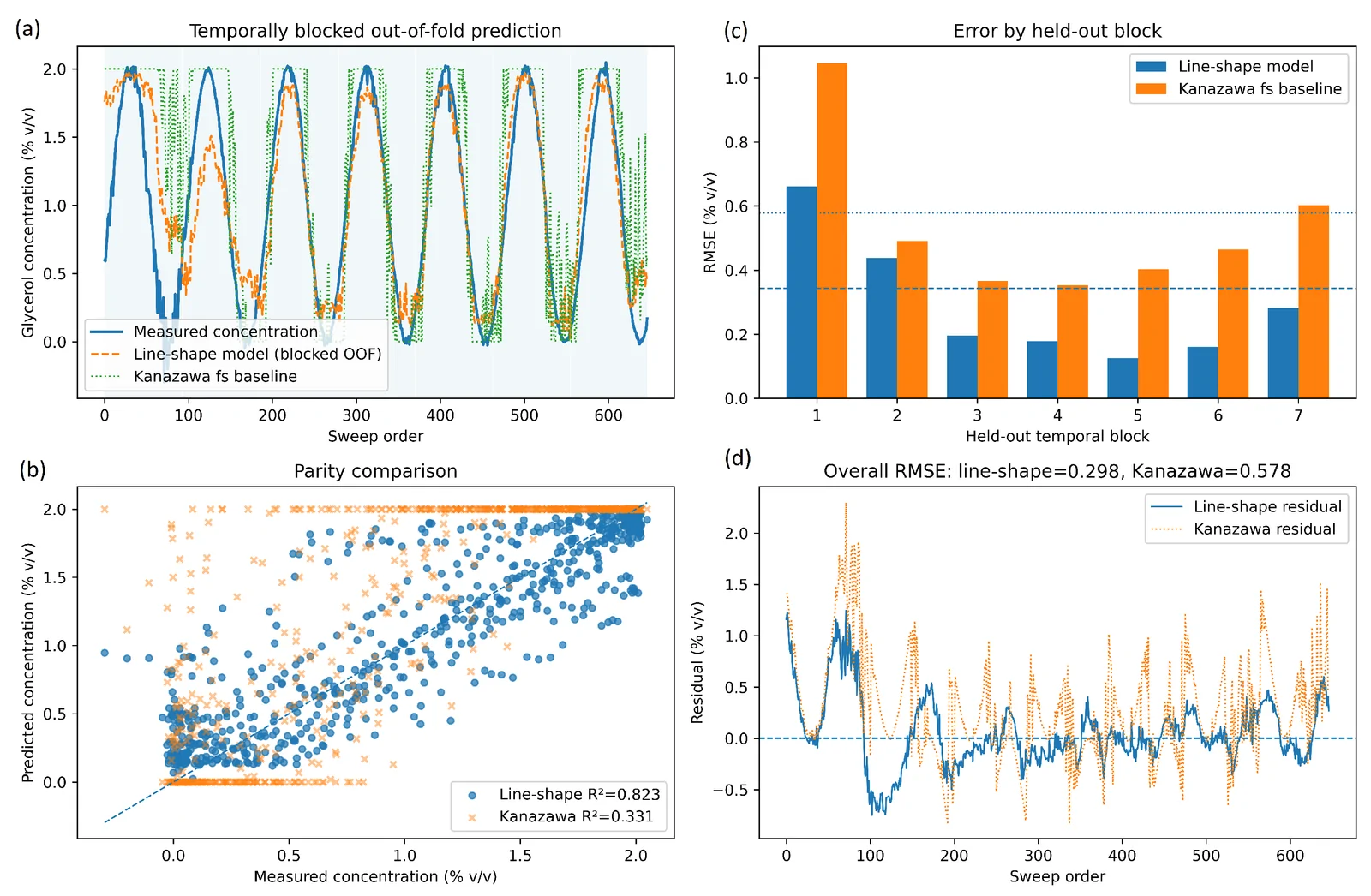
Temporally blocked out-of-fold comparison of the best-performing impedance line-shape model and the row-matched Kanazawa baseline. The line-shape model was a random forest using the top-11 mRMR-MID-ranked descriptors. (**a**) Measured glycerol concentration and out-of-fold predictions from the line-shape model and Kanazawa baseline plotted against sweep order. (**b**) Predicted versus measured concentrations for both approaches; the dashed diagonal denotes ideal agreement. (**c**) RMSE of each approach for each of the seven held-out temporal blocks; the horizontal dashed lines indicate the corresponding overall RMSE values. (**d**) Prediction residuals from both approaches plotted against sweep order; the horizontal dashed line denotes zero residual.

Under identical post-QC sampling, the row-matched Kanazawa baseline yielded $R^{2}=0.331$, RMSE=0.578%v/v, and MAE=0.416%v/v, whereas the selected temporally blocked line-shape model achieved $R^{2}=0.823$, RMSE=0.298%v/v, and MAE=0.217%v/v. These results correspond to reductions of 48.4% in RMSE and 47.8% in MAE relative to the Kanazawa baseline. The largest line-shape prediction error occurred in the first temporal block, which represents a startup/edge-extrapolation case. After this initial interval, the line-shape model followed the imposed concentration waveform more closely and generally produced smaller residuals than the scalar Kanazawa estimate (Figure 6).

**Figure 7:**
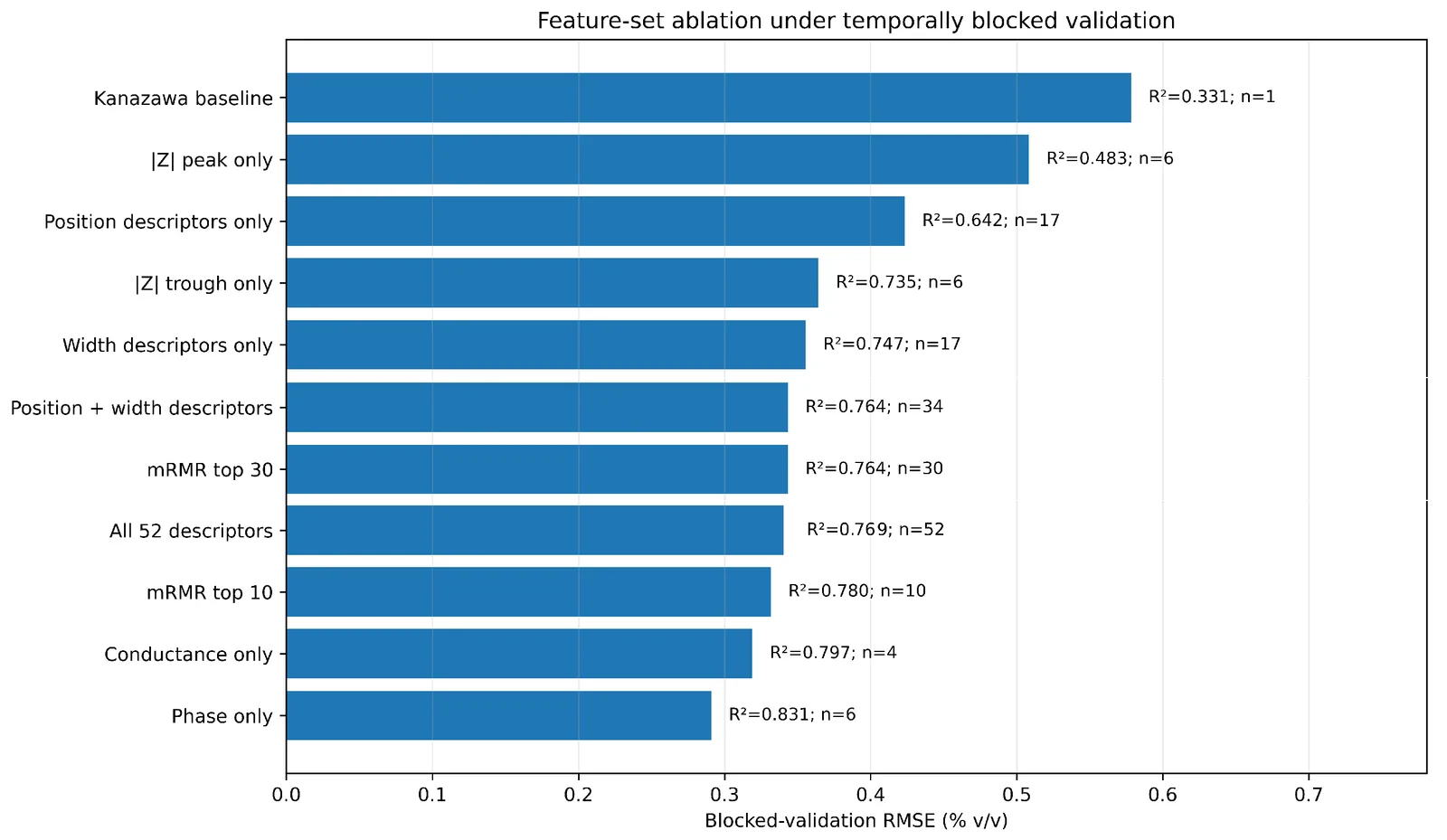
Feature-set ablation under temporally blocked validation. Restricted descriptor families were compared with the row-matched Kanazawa baseline using the same seven-block out-of-fold protocol. Phase-angle and conductance line-shape descriptors contained particularly strong temporally generalizable information, while compact mRMR-selected subsets and the full 52-descriptor representation remained clearly better than the scalar Kanazawa baseline.

The ablation analysis further clarifies which portions of the impedance line shape carry predictive information (Figure 7). Phase descriptors alone achieved $R^{2}=0.831$, RMSE=0.291%v/v, and MAE=0.224%v/v, while conductance descriptors alone achieved $R^{2}=0.797$, RMSE=0.319%v/v, and MAE=0.223%v/v. The mRMR top-10 subset achieved $R^{2}=0.780$ and RMSE=0.332%v/v, and the proposed top-eight subset achieved $R^{2}=0.823$ and RMSE=0.298%v/v, while larger subsets fell slightly below the plateau ($R^{2}=0.764$ at k=30).

### 3.6. Experimental DNA Hybridization Validation

**Figure 8:**
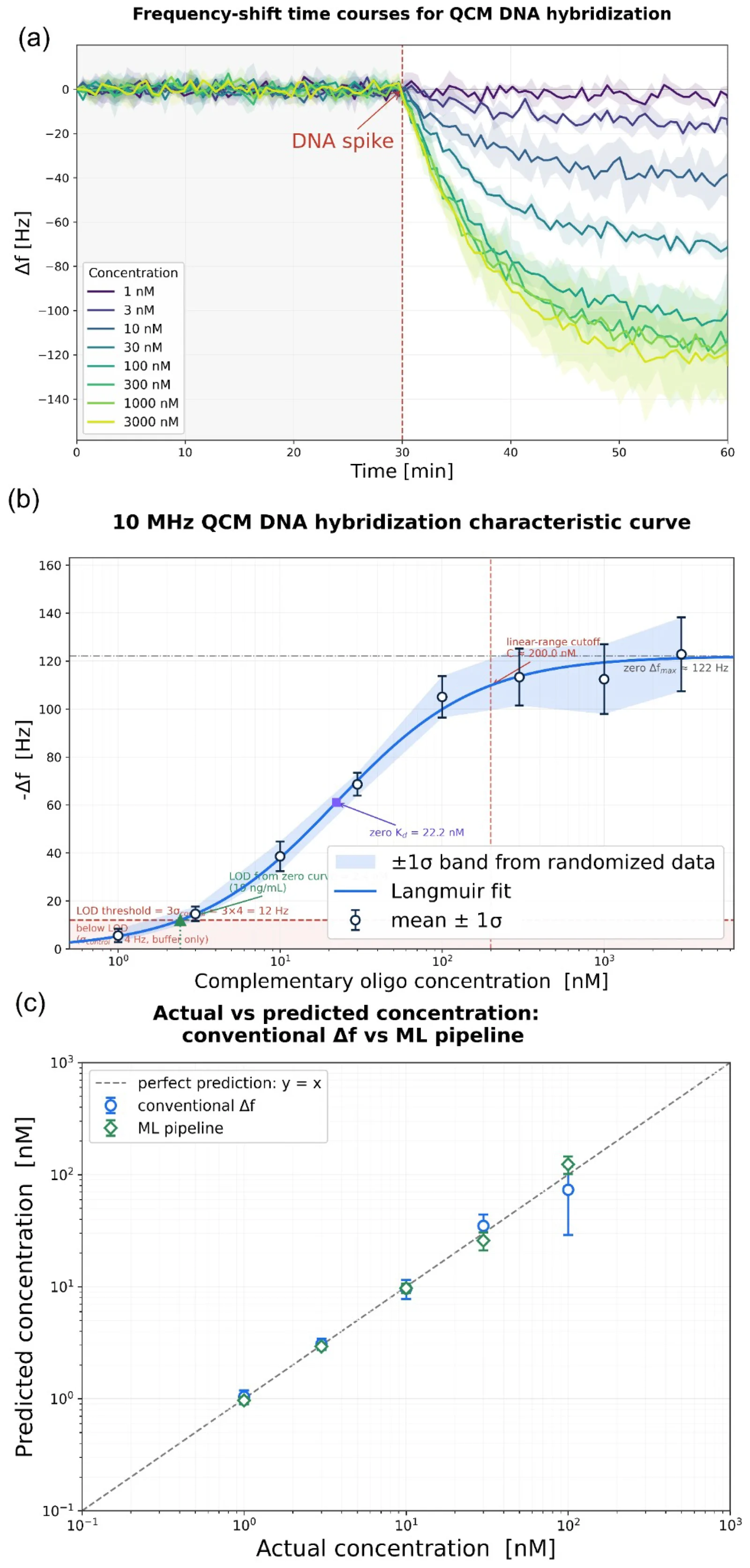
DNA hybridization QCM biosensing validation of the impedance-resolved ML pipeline. (**a**) Frequency-shift time courses recorded before and after target-DNA spiking into 1× PBS at increasing complementary oligonucleotide concentrations. Shaded regions indicate replicate variability (±1σ). (**b**) Experimental QCM DNA hybridization characteristic curve fitted with a Langmuir-type model. The LOD threshold was defined as 3σ of the buffer/control baseline, and the linear-range cutoff used for conventional Δf-based concentration prediction is indicated. (**c**) Actual versus predicted concentration obtained using conventional Δf readout and the impedance line-shape ML pipeline. The dashed line indicates ideal prediction (y=x). Prediction errors were evaluated using all replicate-level concentration predictions rather than only the plotted mean values; the displayed ML comparison uses the conservative uncertainty setting corresponding to 50% of the conventional reference uncertainty.

The glycerol–water experiment establishes that impedance-resolved QCM line-shape descriptors can recover controlled composition changes under constant-flow liquid loading. For biosensor translation, however, the more relevant question is whether the same analysis logic remains useful when the signal originates from a biochemical recognition process. Therefore, an experimental DNA hybridization validation was included as an application-oriented biosensing extension (Figure 8). The time-course data show a stable buffer baseline before target addition and concentration-dependent negative frequency shifts after the DNA spike at 30 min (Figure 8a). The resulting characteristic curve follows a saturating Langmuir-type response, with a fitted apparent $K_{d}$ of 22.2 nM and a fitted maximum frequency shift of approximately 122 Hz (Figure 8b).

Beyond its biosensing relevance, this assay also tests the descriptor set under a structurally different input. The glycerol–water experiment imposes a continuous concentration profile, so a mapping could, in principle, exploit the smooth temporal variation of the target rather than the resonance morphology itself; the temporally blocked protocol of Section 3.5 was applied to control for exactly that. The hybridization series is stepwise, with each concentration separated by an injection transient and preceded by a stable buffer interval, and therefore offers no continuum to interpolate along. That the same line-shape descriptors support quantification in both regimes indicates that the recovered information is a property of the resonance morphology rather than of the concentration schedule.

In the actual-versus-predicted concentration analysis, the conventional Δf calibration was compared with the impedance line-shape ML pipeline using all the replicate-level predictions rather than concentration-wise means. This distinction is important because error bars reflect replicate variability, and reducing each concentration to a single mean would underestimate the prediction-level uncertainty. Across the plotted concentration range, the conventional Δf method produced an MAPE of 21.02%, whereas the ML pipeline produced an MAPE of 10.73%. This corresponds to a 48.9% reduction in replicate-level concentration prediction error. The root mean square percentage error was also lower for the ML pipeline (16.62%) than for conventional Δf prediction (29.24%). These results support the central premise of the study: retaining and learning from impedance line-shape morphology can improve quantitative QCM biosensing beyond scalar frequency-shift calibration.

### 3.7. Non-Specific Binding and Matrix Robustness

The preceding section shows that the descriptor set transfers across two structurally different concentration schedules. Transfer across a change of matrix is a separate question and one the present data do not address. All the DNA hybridization results reported here were obtained in 1× PBS using synthetic oligonucleotides. This choice isolates the contribution of the line-shape representation from matrix effects, but it also means that the reported performance does not extend, without further validation, to complex biological samples such as serum, whole blood, or saliva. Non-specific adsorption in such matrices perturbs the interfacial layer and would be expected to displace the resonance in the same direction as the target-induced response, altering the fitted line-shape descriptors as well as the scalar frequency shift.

Whether the multi-parameter representation confers an advantage under these conditions is an open question, but the mechanistic basis for expecting one is worth stating. The perturbations that confound quantification in real samples do not act on the resonance in the same way. Specific hybridization forms a thin comparatively rigid duplex layer whose signature is dominated by mass loading. Non-specific protein adsorption produces a soft hydrated adlayer that couples strongly to the dissipative descriptors. Variation in ionic strength and pH alters the electrical double layer together with the conformation and charge of the immobilized probe, affecting the conductance and susceptance parameters. Temperature displaces the resonance through the quartz temperature coefficient while simultaneously changing bulk viscosity, producing a joint displacement of the frequency and damping descriptors that is distinct from either effect alone. A scalar Δf readout collapses all of these onto a single axis, and no downstream processing can recover what the reduction discards. A representation that retains the line shape observes them jointly and therefore retains, in principle, the structure required to distinguish them. The present dataset cannot test this: it contains no fouling challenge and no systematic variation in pH, ionic strength or temperature.

Several mitigation strategies follow. Antifouling surface chemistry—oligoethylene glycol backfilling or zwitterionic monolayers—reduces adsorption at source. Differential referencing against a non-complementary probe channel measured in parallel provides a per-experiment estimate of the non-specific contribution, and calibrating frequency shift against concentration on such a channel is the natural next step in establishing that the measured response reflects specific recognition rather than a mixture of specific and non-specific binding. On-chip temperature monitoring with descriptor-level correction addresses the thermal contribution directly. Where these are insufficient, matrix-specific recalibration accepts that the mapping is matrix-dependent and re-estimates it, and perturbed conditions can be included in the training distribution so that the model is fitted across the variation it must tolerate.

The scope of the present contribution should be stated plainly. What is established here is a representation—a dense parameterization of resonance morphology—together with a validation protocol that is strict enough to show that the mapping estimated from it generalizes beyond the interval over which it was fitted. The questions that follow are substantial and largely open: how the noise structure of the descriptors behaves under fouling and environmental perturbation, whether limit of detection admits a multivariate definition that is appropriate to a high-dimensional readout rather than a scalar one, and how the descriptor set behaves across matrices and recognition chemistries. These are directions this work is intended to open rather than to close, and each requires experimental designs beyond the two reported here.

## 4. Conclusions

Impedance-resolved QCM, integrated with a passive microfluidic mixer and a redundancy-aware machine-learning pipeline, was shown to enable temporally robust prediction of small composition changes in the 0–2% (*v*/*v*) glycerol range. By converting each sweep of nine spectra into 52 physically interpretable line-shape features and applying consensus outlier handling followed by training-fold mRMR ranking, the workflow achieved high shuffled five-fold performance ($R^{2}=0.948$, RMSE=0.162%v/v) and retained predictive value under the stricter seven-block temporal validation protocol ($R^{2}=0.823$, RMSE=0.298%v/v). Under the same row-matched comparison, the Kanazawa $f_{s}$ baseline derived from the |Z| trough position yielded $R^{2}=0.331$ and RMSE=0.578%v/v. Thus, the temporally blocked line-shape model reduced RMSE by 48.4% relative to the scalar Kanazawa baseline. These results indicate that preserving full resonance morphology—rather than relying only on scalar Δf or ΔD endpoints—unlocks robust information-rich signals for concentration prediction.

The experimental DNA hybridization validation strengthens the biosensing relevance of the workflow by moving the analysis beyond controlled bulk liquid-property inference to a recognition-based QCM assay. In this setting, the impedance line-shape ML pipeline reduced replicate-level MAPE from 21.02% for conventional Δf prediction to 10.73%, corresponding to a 48.9% reduction in concentration prediction error. This result suggests that impedance-resolved feature extraction can improve concentration estimation in QCM biosensors even when the sensor response is governed by biomolecular binding rather than only Newtonian liquid loading.

From an instrumentation perspective, these findings suggest that QCM workflows constrained to Δf/ΔD-only endpoints can become an information bottleneck for inference tasks. When prediction accuracy is the primary objective, restricting acquisition and analysis to two scalar readouts may be suboptimal compared with multi-observable impedance-resolved line-shape processing. The workflow is model-agnostic and hardware-light, making it readily extensible to additional analytes, viscoelastic films, higher overtones, and other piezoelectric resonators provided that appropriate calibration data are available.

The most immediate methodological extension is to multi-harmonic acquisition. The present study confines itself deliberately to the fundamental resonance, this being the most constrained setting in which the central question can be posed: if line-shape parameterization improves quantification on a single harmonic in a Newtonian liquid, the improvement is attributable to the scalar reduction itself rather than the limited observability of the measurement. Multi-harmonic QCM-D enriches the measurement along a different axis since the overtone dependence of the penetration depth carries depth-resolved information about the interfacial layer that no single-harmonic acquisition can supply [53]. The two are therefore complementary rather than alternative, and they compose without modification: the parameterization described here applies independently to the resonance recorded at each harmonic so that the descriptor set scales with the number of harmonics acquired. Such a combination would make redundancy-aware ranking more rather than less important because descriptors derived from neighboring harmonics are expected to be strongly correlated, and it would permit a direct test of whether depth-resolved and shape-resolved information is genuinely independent or substantially redundant. The practical cost is acquisition time: sweeping several narrow windows at each of several harmonics lengthens the measurement cycle and therefore trades against the temporal cadence that makes the present single-harmonic implementation suitable for following a continuously evolving concentration. Establishing where that trade-off is favorable is a well-posed question that the descriptor set defined here is directly suited to answering.

More broadly, the perturbations that confound quantitative biosensing in real samples—non-specific adsorption, ionic strength, pH, and temperature—act on the resonance line shape in mechanistically distinct ways, and a representation that retains that shape retains, in principle, the structure required to distinguish them. Testing whether this separability can be realized in practice is the direction we regard as the most consequential for translating impedance-resolved QCM biosensing beyond controlled buffer conditions. 

## Figures and Tables

**Table 1:** Resonance features and sweep windows (1000 points, no averaging).

Feature	Window Size	Center Frequency
$B_{\mathrm{peak}}$	3 kHz	10008449
$B_{\mathrm{trough}}$	3 kHz	10014600
$Z_{\theta }$	5 kHz	10011947
${\left|Z\right|}_{\mathrm{trough}}$	5 kHz	10008836
${\left|Z\right|}_{\mathrm{peak}}$	5 kHz	10015060
$X_{\mathrm{peak}}$	5 kHz	10009210
$X_{\mathrm{trough}}$	5 kHz	10015444
*R*	3 kHz	10012285
*G*	50 kHz	10011585

**Table 2:** Rank-sorted fit parameters. Columns indicate contiguous rank ranges; rank increases top-to-bottom within each column and then continues in the next column.

Ranks 1–11	Ranks 12–22	Ranks 23–33	Ranks 34–44	Ranks 45–52
$G^{a}$	$Z_{\Theta }^{b1}$	$\underline{{\left|Z\right|}_{\mathrm{peak}}^{a2}}$	$X_{trough}^{a1}$	$X_{trough}^{c1}$
$Z_{\Theta }^{c2}$	$\underline{B_{\mathrm{trough}}^{c1}}$	${\left|Z\right|}_{\mathrm{trough}}^{b2}$	$X_{peak}^{a1}$	$B_{peak}^{b2}$
$G^{b}$	$X_{peak}^{b2}$	${\left|Z\right|}_{\mathrm{trough}}^{c1}$	$\underline{B_{\mathrm{trough}}^{a1}}$	$B_{peak}^{a1}$
$X_{trough}^{c2}$	$\underline{{\left|Z\right|}_{\mathrm{peak}}^{b2}}$	$\underline{{\left|Z\right|}_{\mathrm{peak}}^{b1}}$	$B_{peak}^{c2}$	$Z_{\Theta }^{a2}$
$Z_{\Theta }^{b2}$	${\left|Z\right|}_{\mathrm{trough}}^{c2}$	$X_{peak}^{c1}$	${\left|Z\right|}_{\mathrm{trough}}^{b1}$	$\bar{R^{a1}}$
$X_{peak}^{a2}$	$\underline{B_{\mathrm{trough}}^{b2}}$	$\bar{R^{c2}}$	$\bar{R^{c1}}$	$B_{peak}^{b1}$
$G^{c}$	$X_{peak}^{c2}$	$\underline{B_{\mathrm{trough}}^{a2}}$	$\underline{{\left|Z\right|}_{\mathrm{peak}}^{c1}}$	$X_{trough}^{a2}$
${\left|Z\right|}_{\mathrm{trough}}^{a1}$	$B_{peak}^{a2}$	$X_{trough}^{b2}$	$Z_{\Theta }^{a1}$	$\bar{R^{b1}}$
${\left|Z\right|}_{\mathrm{trough}}^{a2}$	$X_{peak}^{b1}$	$\underline{B_{\mathrm{trough}}^{b1}}$	$B_{peak}^{c1}$	
$Z_{\Theta }^{c1}$	$\underline{B_{\mathrm{trough}}^{c2}}$	$\underline{{\left|Z\right|}_{\mathrm{peak}}^{a1}}$	$\underline{{\left|Z\right|}_{\mathrm{peak}}^{c2}}$	
$\bar{R^{b2}}$	$X_{trough}^{b1}$	$G^{d}$	$\bar{R^{a2}}$	
**Legend: font style → parameter family**				
**Style**	**Family**	**Style**	**Family**	
*G*	*G*	${\left|Z\right|}_{\mathrm{trough}}$	${\left|Z\right|}_{trough}$	
$Z_{\Theta }$	$Z_{\Theta }$	$\underline{{\left|Z\right|}_{\mathrm{peak}}}$	${\left|Z\right|}_{peak}$	
$X_{peak}$	$X_{peak}$	$B_{peak}$	$B_{peak}$	
$X_{trough}$	$X_{trough}$	$\underline{B_{\mathrm{trough}}}$	$B_{trough}$	
$\bar{R}$	*R*			

Exponents represent fit terms; Gaussian fits are two-term (a1,b1,c1,a2,b2,c2); a Lorentzian function with constant offset was fitted on *G*(a,b,c,d).

## Data Availability

The raw impedance datasets generated for the glycerol calibration experiments and the analysis codes used for feature extraction, model training, and machine-learning evaluation will be made available in a public GitHub repository upon acceptance. Due to the ongoing nature of the funded project (Grant No. 122E166) and to protect potential intellectual property, the raw time-series files from the DNA hybridization assay are temporarily restricted. However, the experimental conditions, processed results, and the complete analysis workflow supporting the DNA hybridization results are fully reported in the article and Supplementary Materials to ensure methodological transparency.

## Supplementary Materials

The following supporting information can be downloaded at: https://www.mdpi.com/article/10.3390/bios16100574/s1. Additional experimental details, supplementary figures, and supplementary tables are provided in the manuscript_supplementary.docx.
