# Supplementary material for: Temporally Robust Machine-Learning-Assisted Impedance Line-Shape Analysis for Quantitative QCM Biosensing with DNA Hybridization Validation
Source: Biosensors (Basel). 2026 Oct 12;16(10):574. doi: 10.3390/bios16100574 (PMC13702575; doi:10.3390/bios16100574)

Supplementary Material:

S.1. Fabrication of microfluidic passive mixer and flow cell

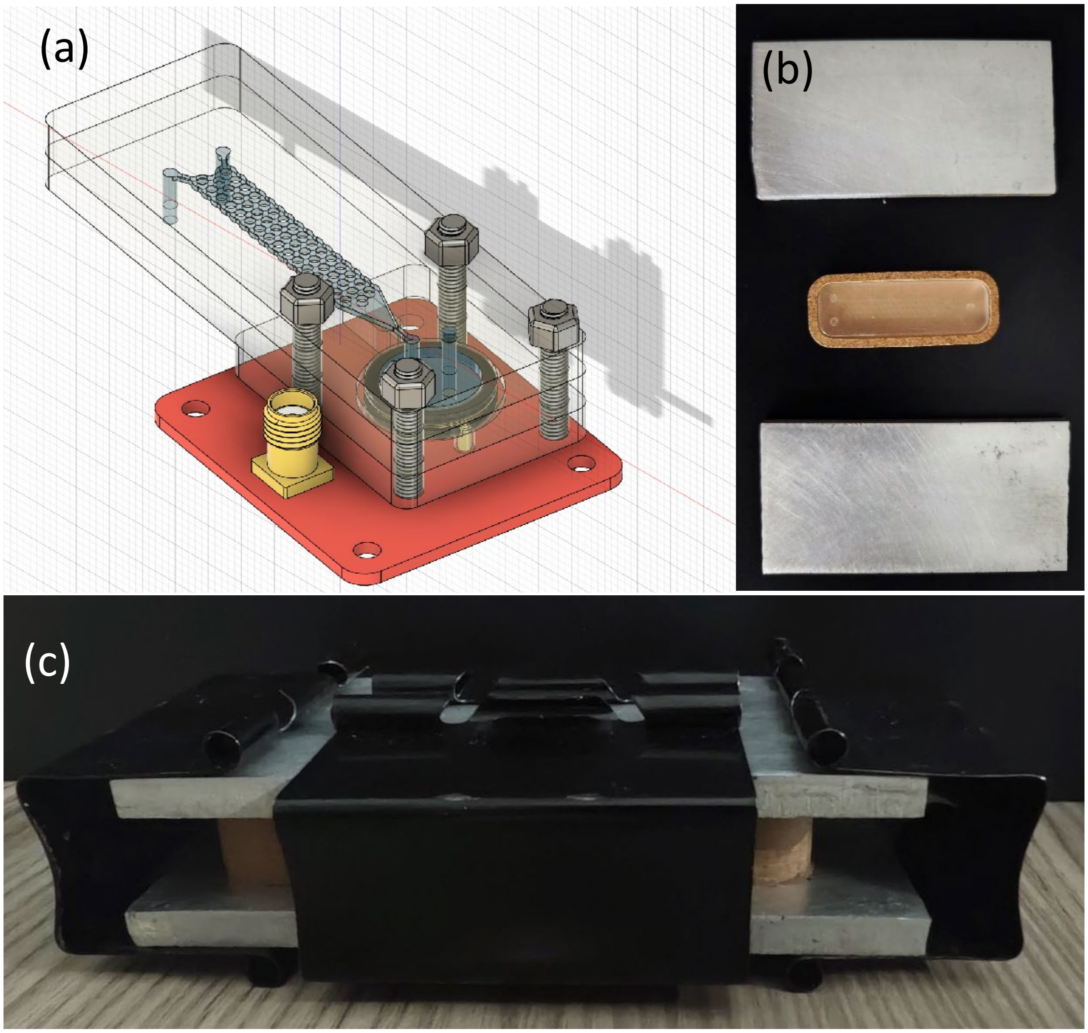

(a) Fusion design of flow cell integrated with microfuidic passive mixer on PCB. SMA connector is not used this experiment. Pogo pins are directly attached to the impedance analyzer fixture. (b) Setup used to thermally fusing the top two acrylic pieces together. An mdf housing is CNC cut and sandwiched in between two 6 mm thick aluminium plates. (c) Clips used to generate the required force for fusing the acrylic pieces is shown. This piece was heated inside the furnace as explained in the materials and methods.

S.2. Gaussian and Laurentzian Functions

A. Fitting parameters of functions

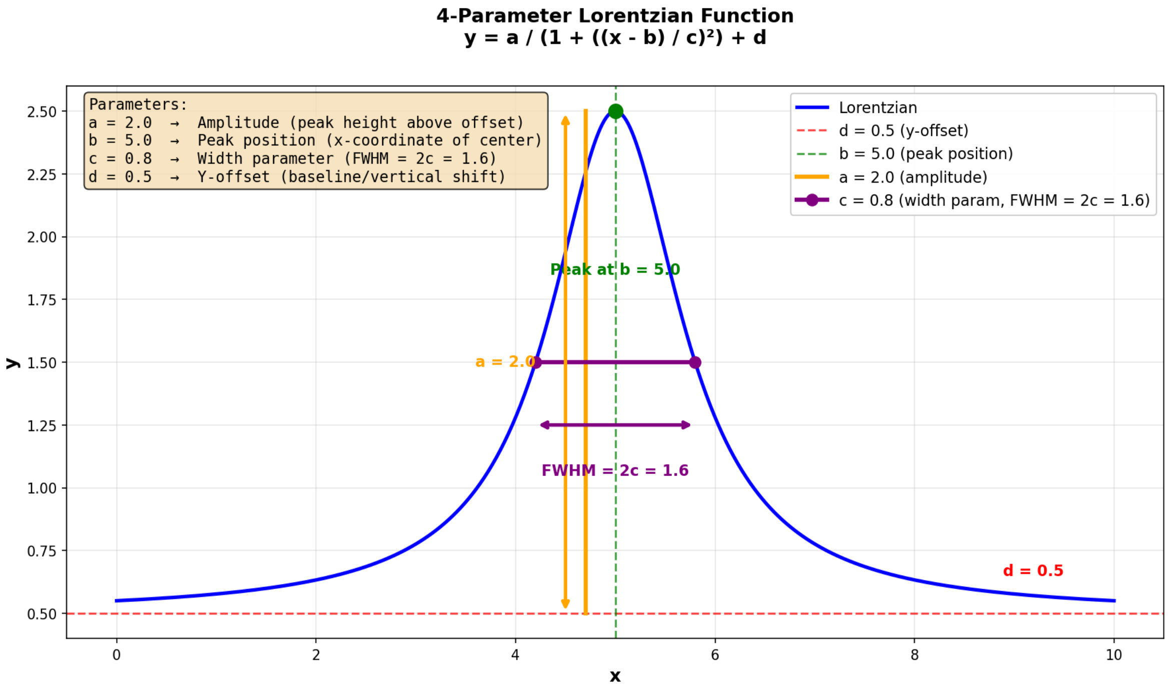

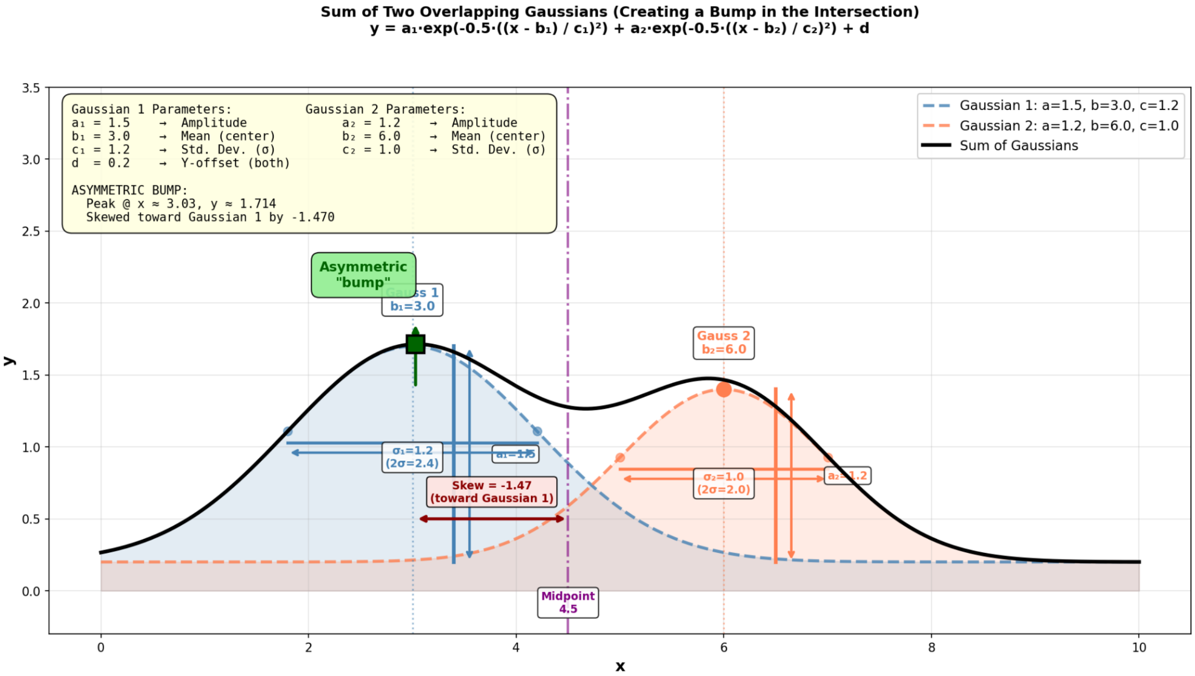

B. Unbounded overfitting example

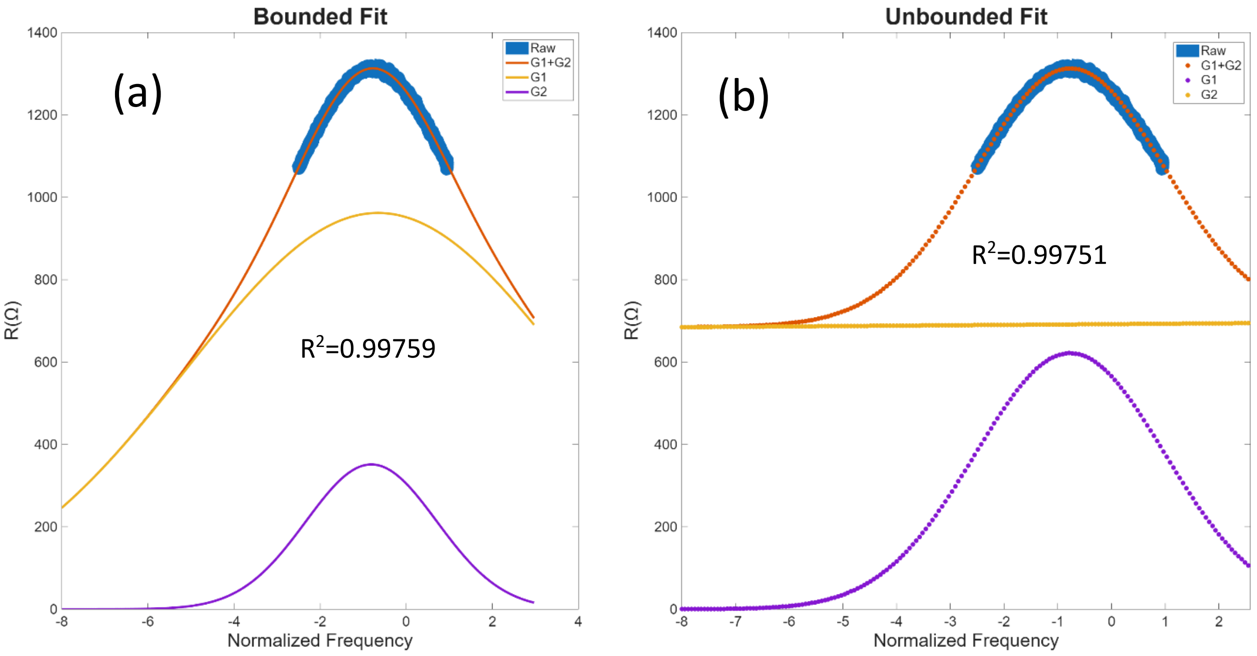

(a) Bounded and (b) unbounded fits are plotted. G1 and G2 represents the Gaussian terms fitted on raw data (blue) for a resistance sweep. G1+G2 represents the final two-term Gaussian fit. An increase of 0.00004 increase was observed in R^2 when no boundaries are set for fittings.

S.3. Simulated impedance spectra

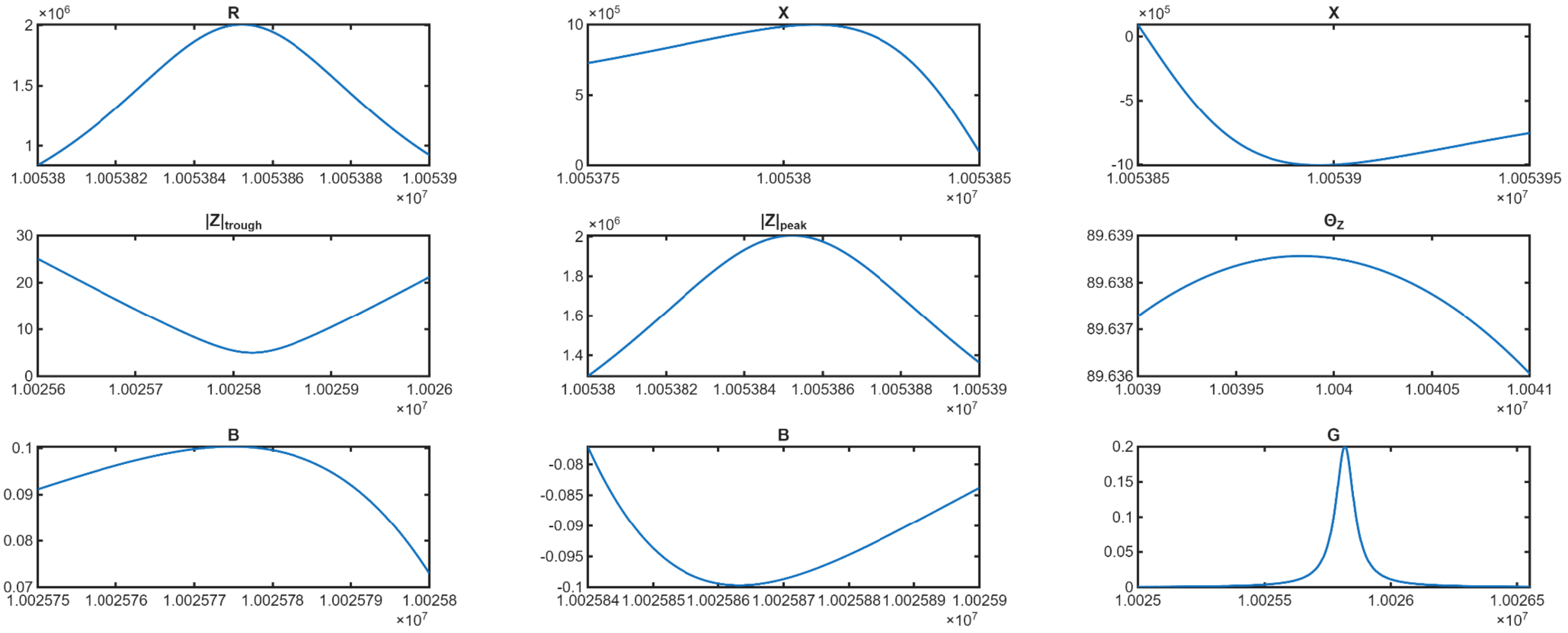

S.4. Bivariate Results

HSIC vs Concentration (All Features; bars colored by impedance parameter group)

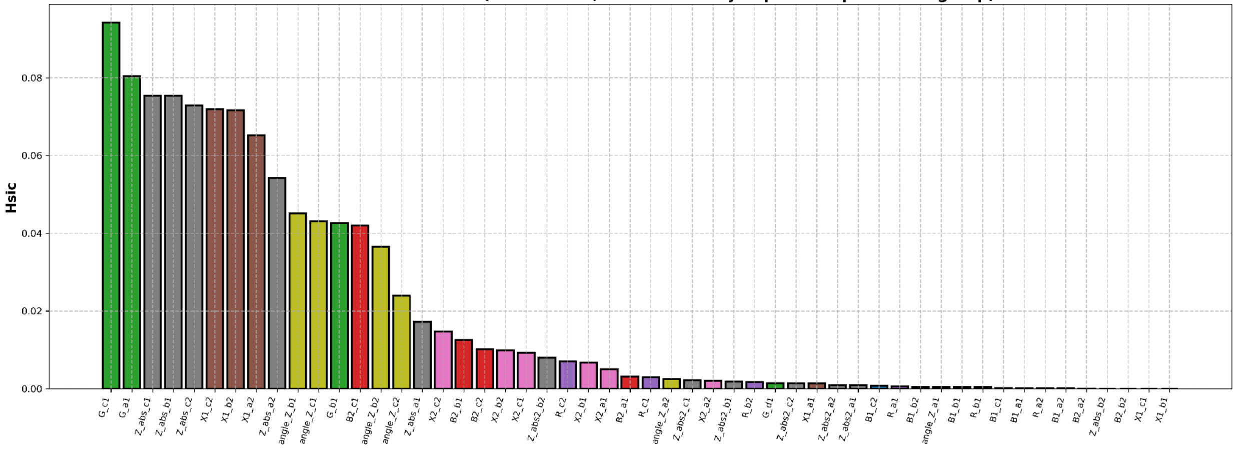

Mutual Information vs Concentration (All Features; bars colored by group)

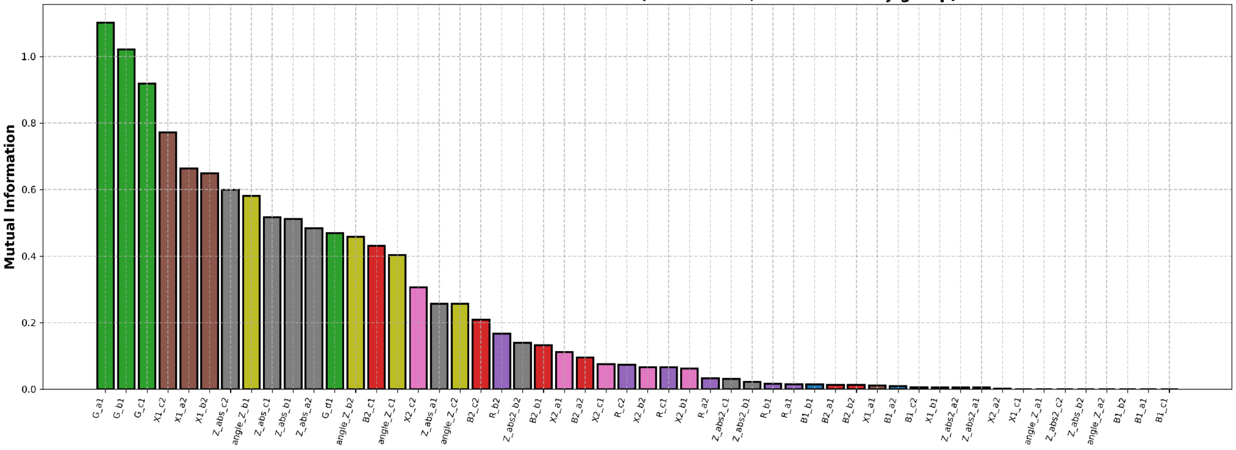

Pearson Correlation (signed r) vs Concentration (All Features; colored by group)

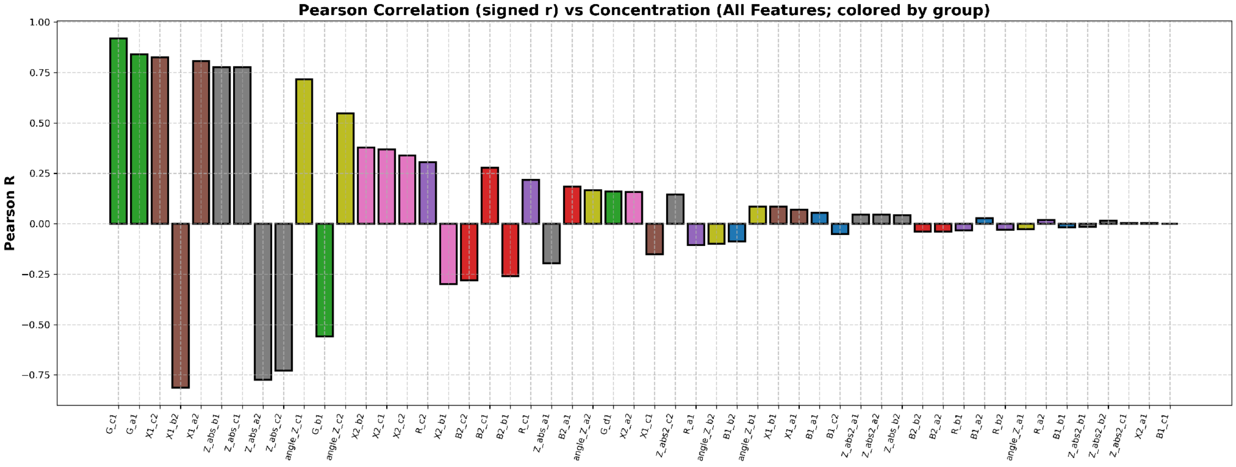

S.5. Outlier Removal

Local Distance-based Outlier Factor (LDOF). LDOF detects points that are unusually far from their *local* neighbourhood relative to the neighbourhood's own spread. For each sample, we find its k-nearest neighbours and compute (i) the mean distance from the sample to those neighbours and (ii) the mean pairwise distance among the neighbours themselves. The LDOF score is the ratio of these two quantities; values near 1 indicate typical points, while larger values indicate local outliers (the sample is much farther from its neighbours than they are from each other). We standardise features before distance computations, use a moderate k(e.g., 15–25), and flag outliers by a robust threshold (high percentile or median + MAD rule). This yields a simple, scale-aware detector that is sensitive to boundary deviations without assuming a global distribution.

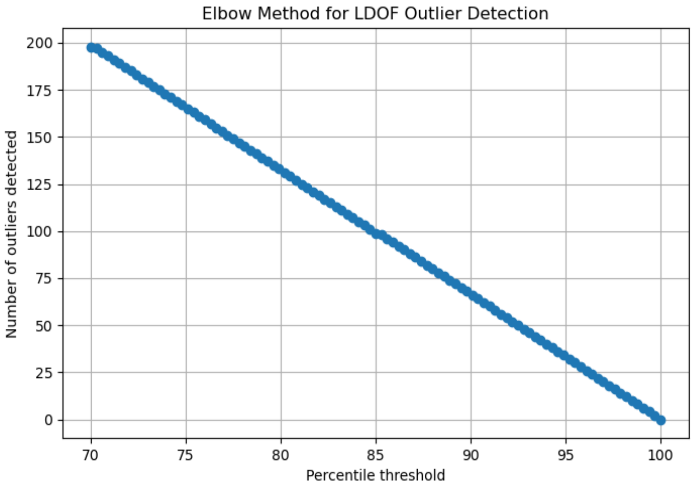

No elbow values are observed for LDOF method.

Isolation Forest (IF). IF identifies anomalies by randomly partitioning the feature space and measuring how quickly a point becomes isolated. An ensemble of binary trees is grown from subsamples; at each node, a random feature and split value partition the data. For a given sample, the average path length across trees provides an anomaly score: shorter paths ⇒ easier to isolate ⇒ more anomalous. IF makes no parametric distributional assumptions, handles high-dimensional data, and is robust to irrelevant features through random subspace splits. Key settings include the number of trees (*n_estimators*), subsample size (*max_samples*), and target outlier proportion (*contamination*) to derive a decision threshold. In practice, we convert path lengths to a continuous score, normalize across runs, and set a threshold via a robust rule (e.g., high percentile or elbow), thereby flagging points with consistently short isolation paths as outliers.

No elbow values are observed for IF method.

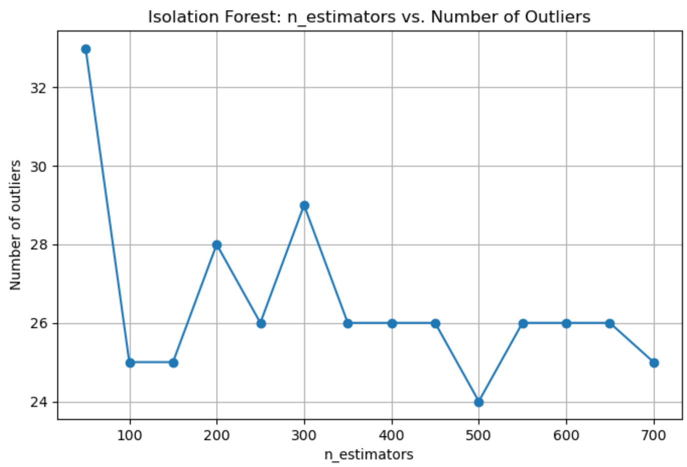

Mahalanobis Distance (MD). MD flags observations that are far from the multivariate center after accounting for feature covariance. For each sample, the distance to the mean is measured in the metric induced by the inverse covariance matrix, so deviations along low-variance (highly constrained) directions count more than those along high-variance directions. Under an approximately Gaussian, elliptic data cloud, the squared MD follows a χ^2distribution with d degrees of freedom, enabling a principled threshold). In practice, we standardise features and estimate covariance with a robust shrinkage method to stabilise the inverse in the presence of correlation or limited samples. Points exceeding the threshold are labelled outliers, capturing globally atypical observations that depart from the dominant elliptical structure.

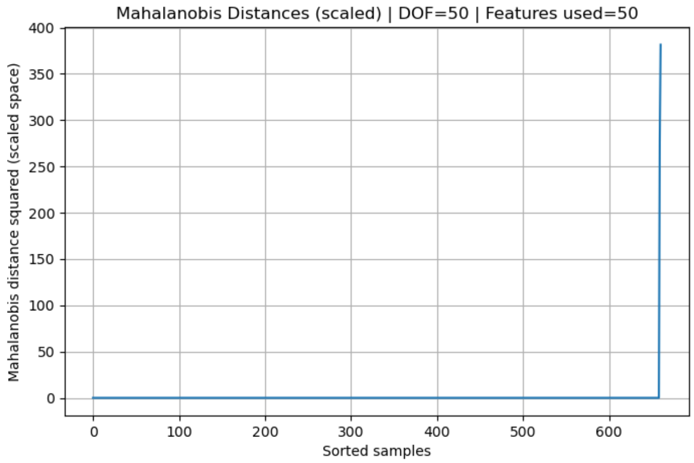

No elbow is observed for the MD method.

S.6. PCA results

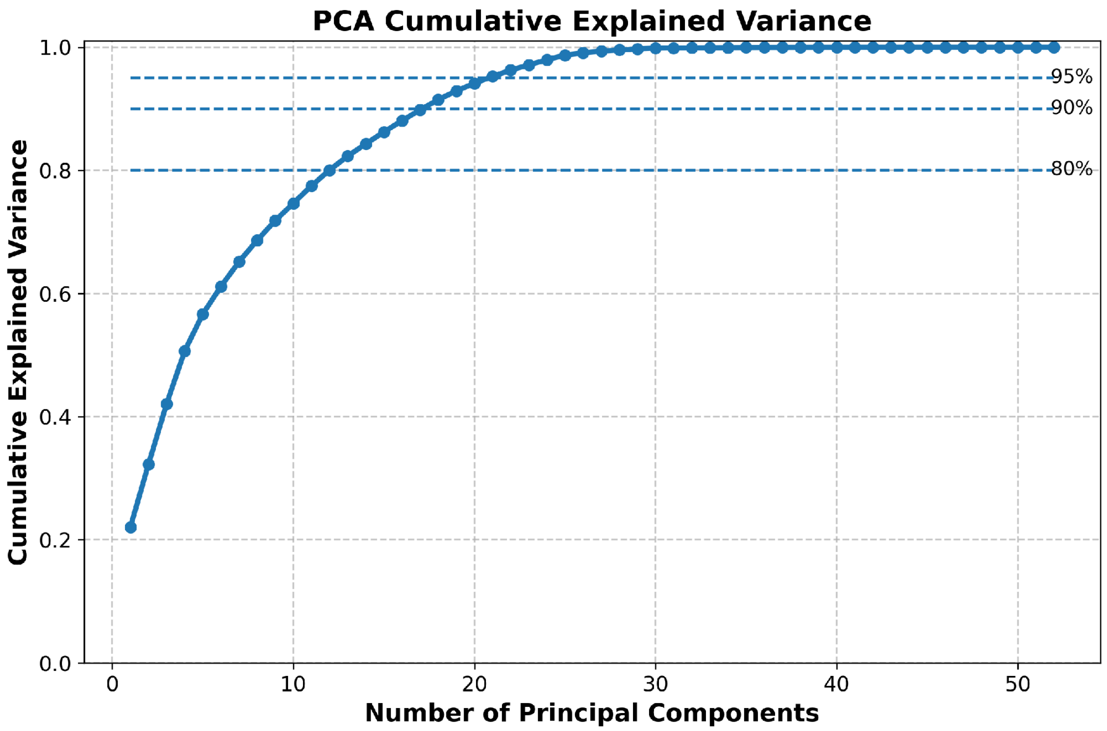

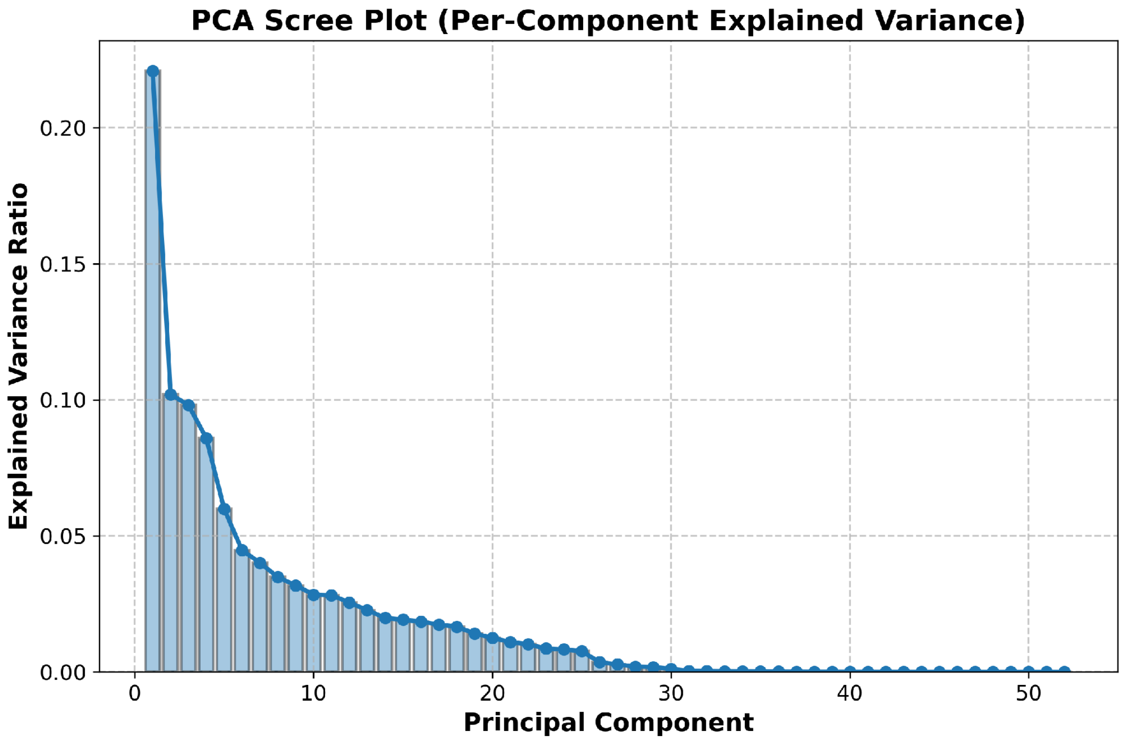

S.7. mRMR-MID Ranking

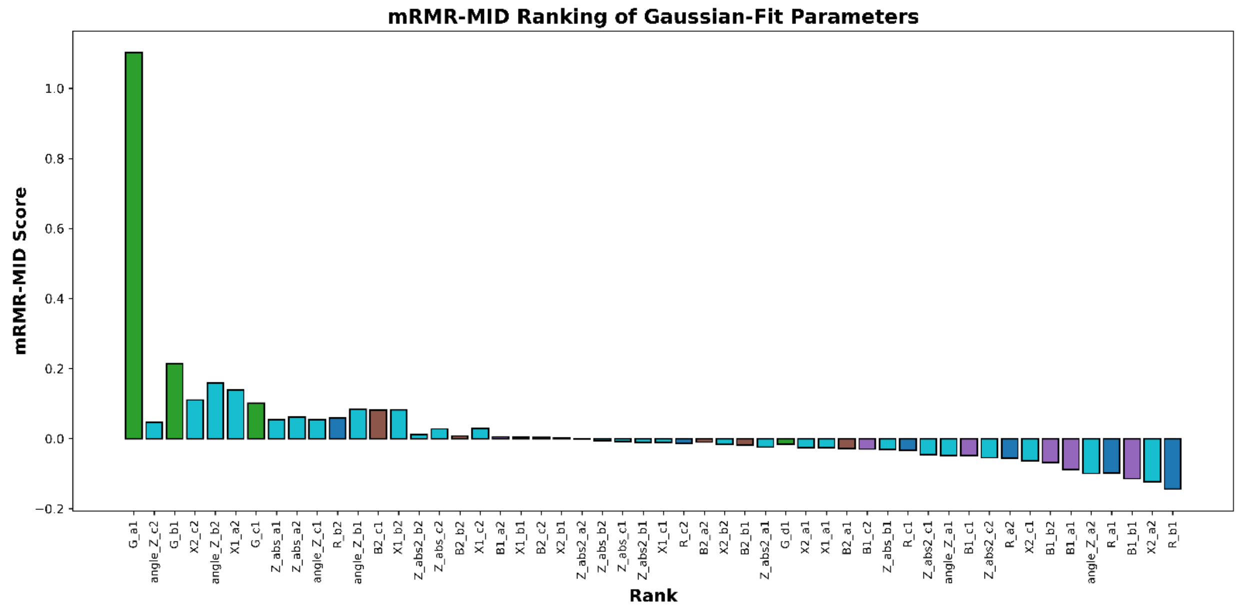

mRMR–MID ranking (how to read the scores).

In the minimum-redundancy maximum-relevance (mRMR) framework with the MID criterion (relevance minus redundancy), the score assigned to a candidate feature at step $t$depends on the features already selected at steps $1, \ldots, t-1$. Consequently, MID values are path-dependent and not on a common scale across steps; a bar plot of raw MID scores is therefore not numerically comparable from one rank position to the next. The ordering—i.e., which feature is chosen at each step—is the meaningful output, while the absolute heights primarily reflect transient trade-offs between added relevance and incremental redundancy at that moment. For reporting, emphasize the rank order (and downstream validation such as R^2vs. top-k), and, if desired, assess robustness via rank stability across bootstrap resamples rather than by comparing MID magnitudes.

S.8. Regression Model Results

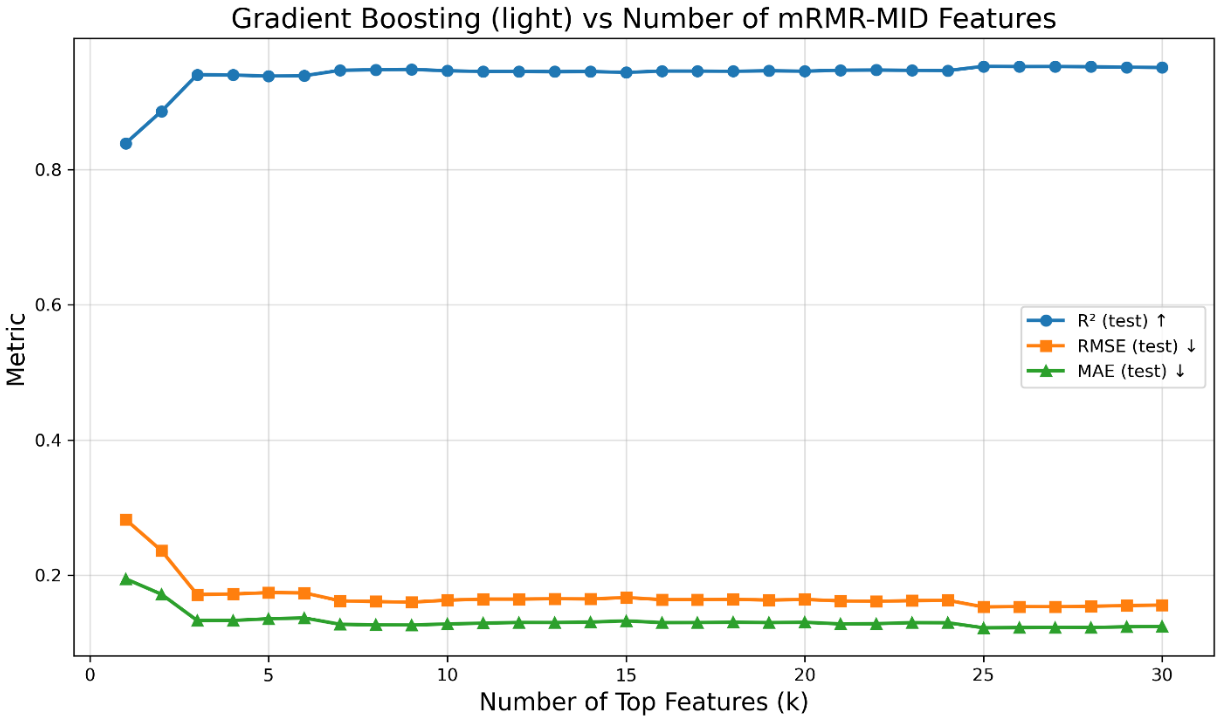

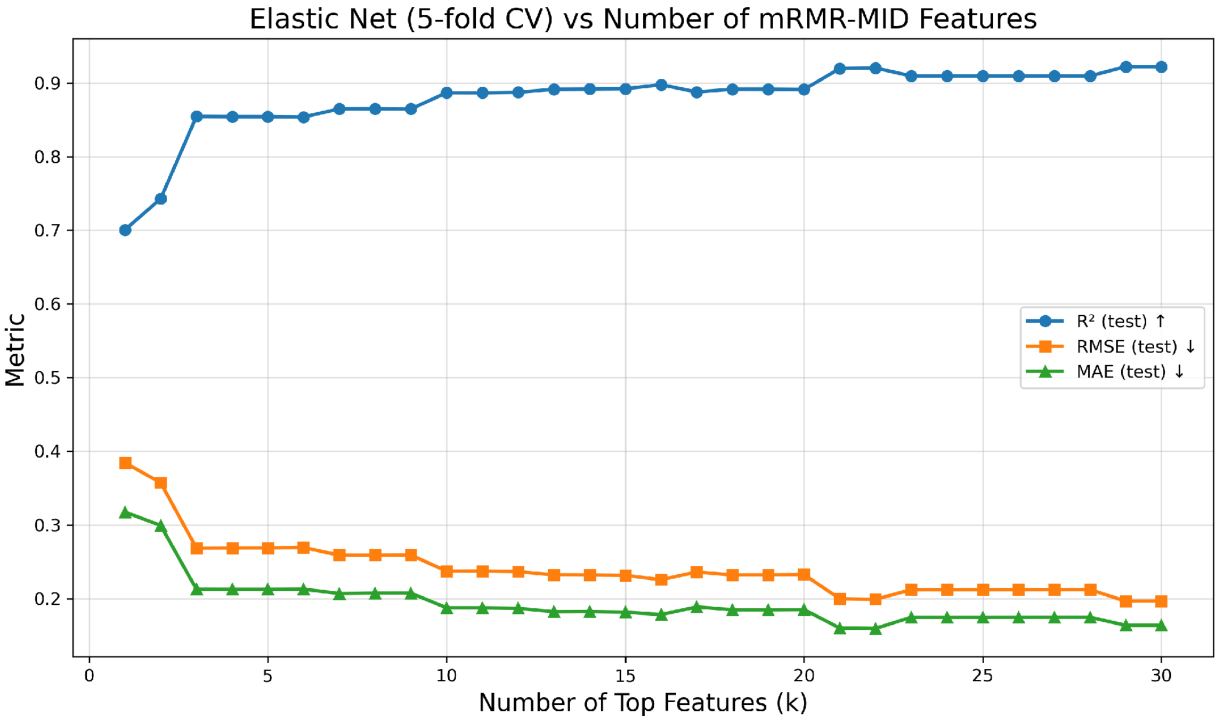

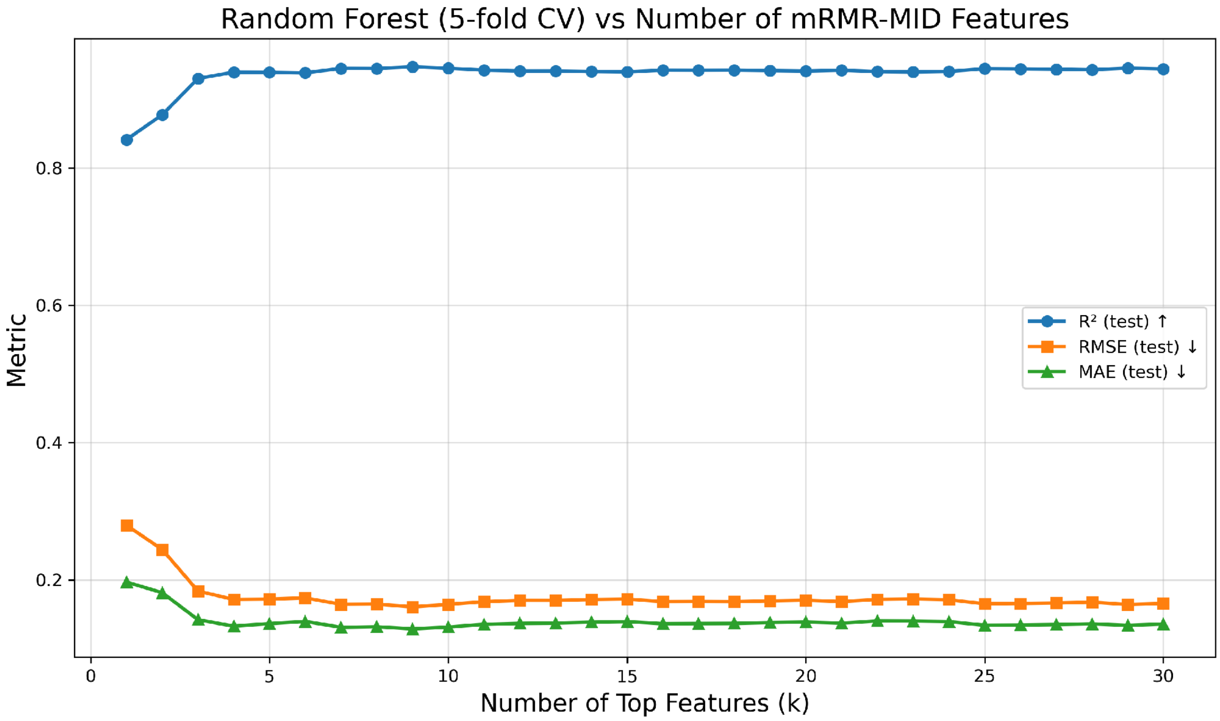
Random Forest (5-fold CV) vs Number of mRMR-MID Features
Metric
Number of Top Features (k)
R² (test) ↑
RMSE (test) ↓
MAE (test) ↓
0.8
0.6
0.4
0.2
0
5
10
15
20
25
30

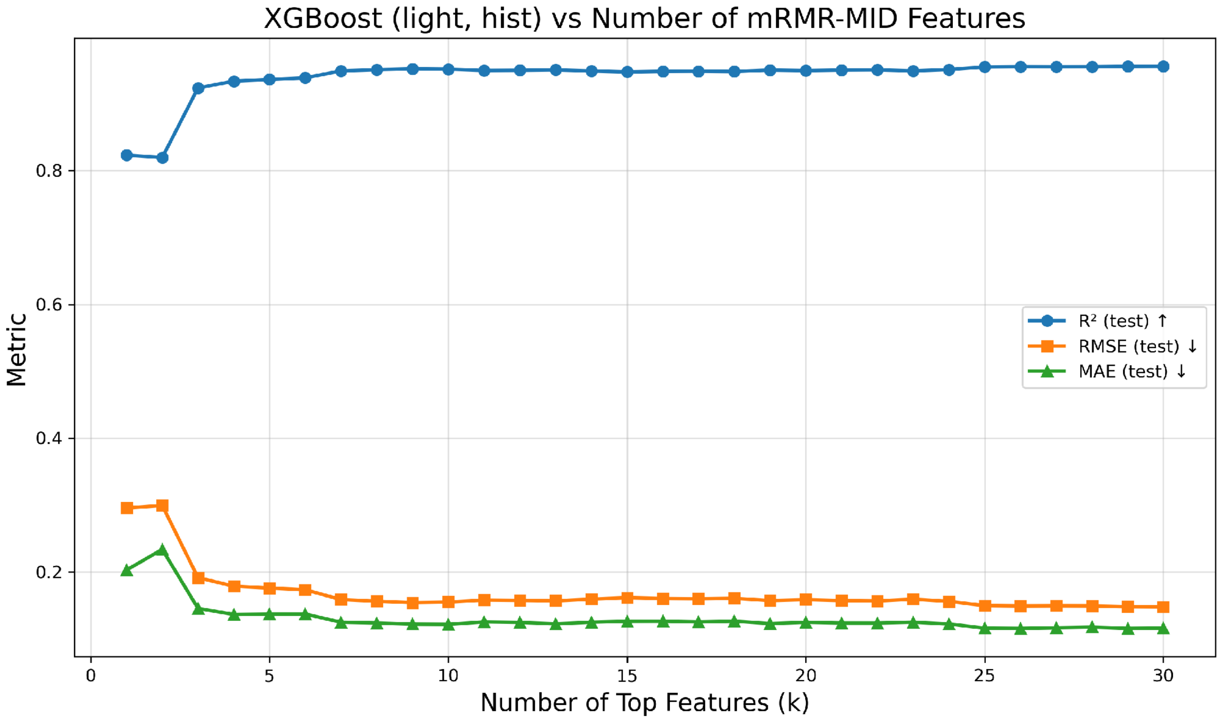
XGBoost (light, hist) vs Number of mRMR-MID Features
Metric
Number of Top Features (k)
R² (test) ↑
RMSE (test) ↓
MAE (test) ↓
0.8
0.6
0.4
0.2
0
5
10
15
20
25
30

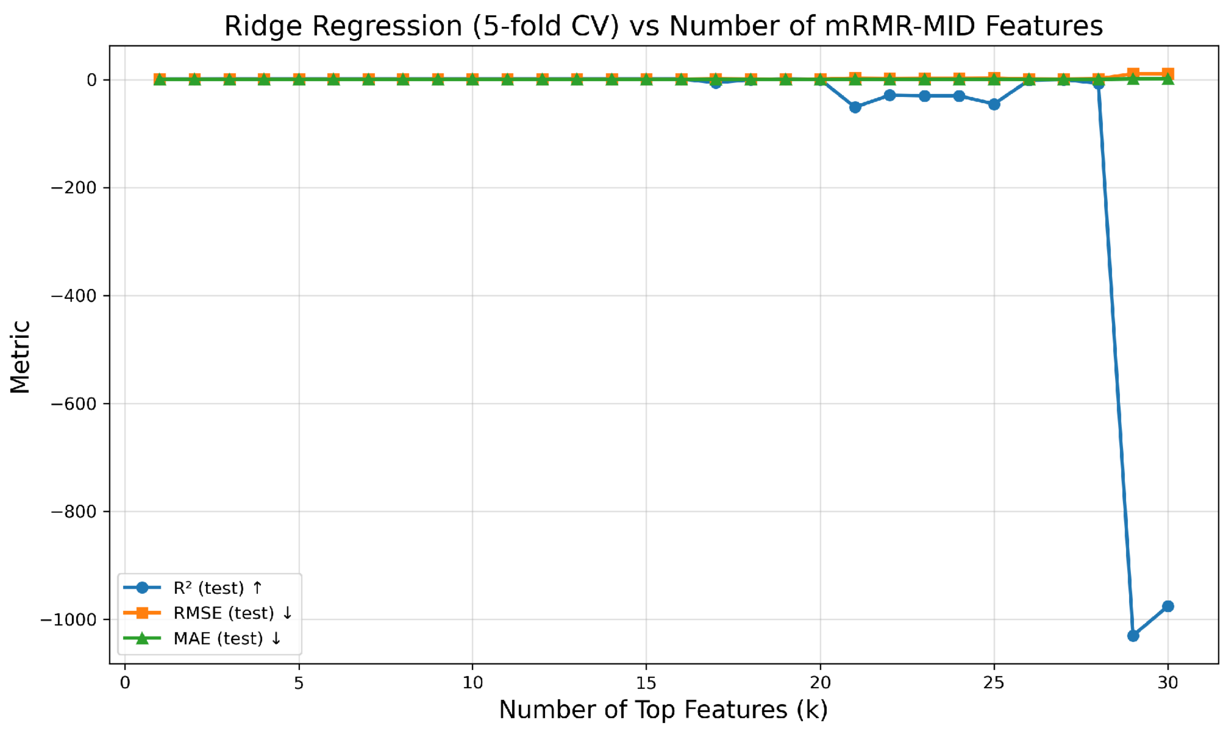

Ridge Regression (5-fold CV) vs Number of mRMR-MID Features
Metric
Number of Top Features (k)
0
−200
−400
−600
−800
−1000
0
5
10
15
20
25
30
R² (test) ↑
RMSE (test) ↓
MAE (test) ↓

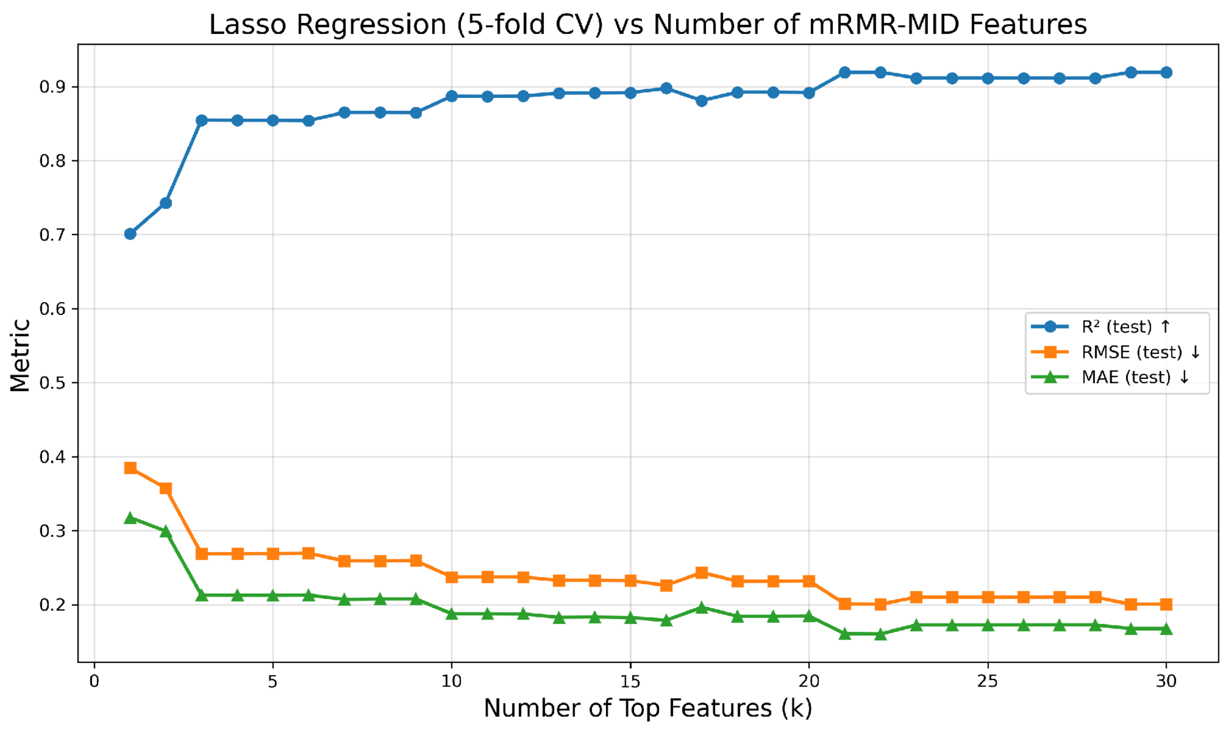

Lasso Regression (5-fold CV) vs Number of mRMR-MID Features
Metric
Number of Top Features (k)
0.9
0.8
0.7
0.6
0.5
0.4
0.3
0.2
0
5
10
15
20
25
30
R² (test) ↑
RMSE (test) ↓
MAE (test) ↓

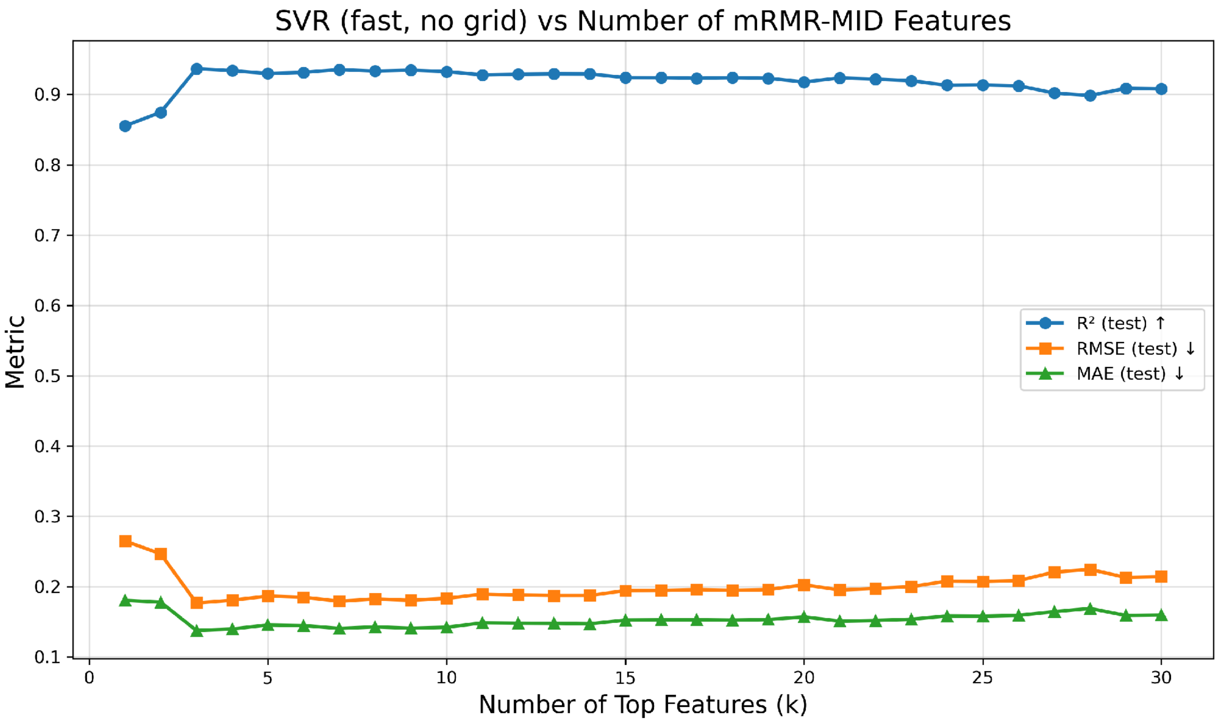

SVR (fast, no grid) vs Number of mRMR-MID Features
Metric
Number of Top Features (k)
R² (test) ↑
RMSE (test) ↓
MAE (test) ↓
0.1
0.2
0.3
0.4
0.5
0.6
0.7
0.8
0.9
0
5
10
15
20
25
30

S.9. Oscillatory Behaviour in Fit Parameters (plotted between 2-98 percentile)

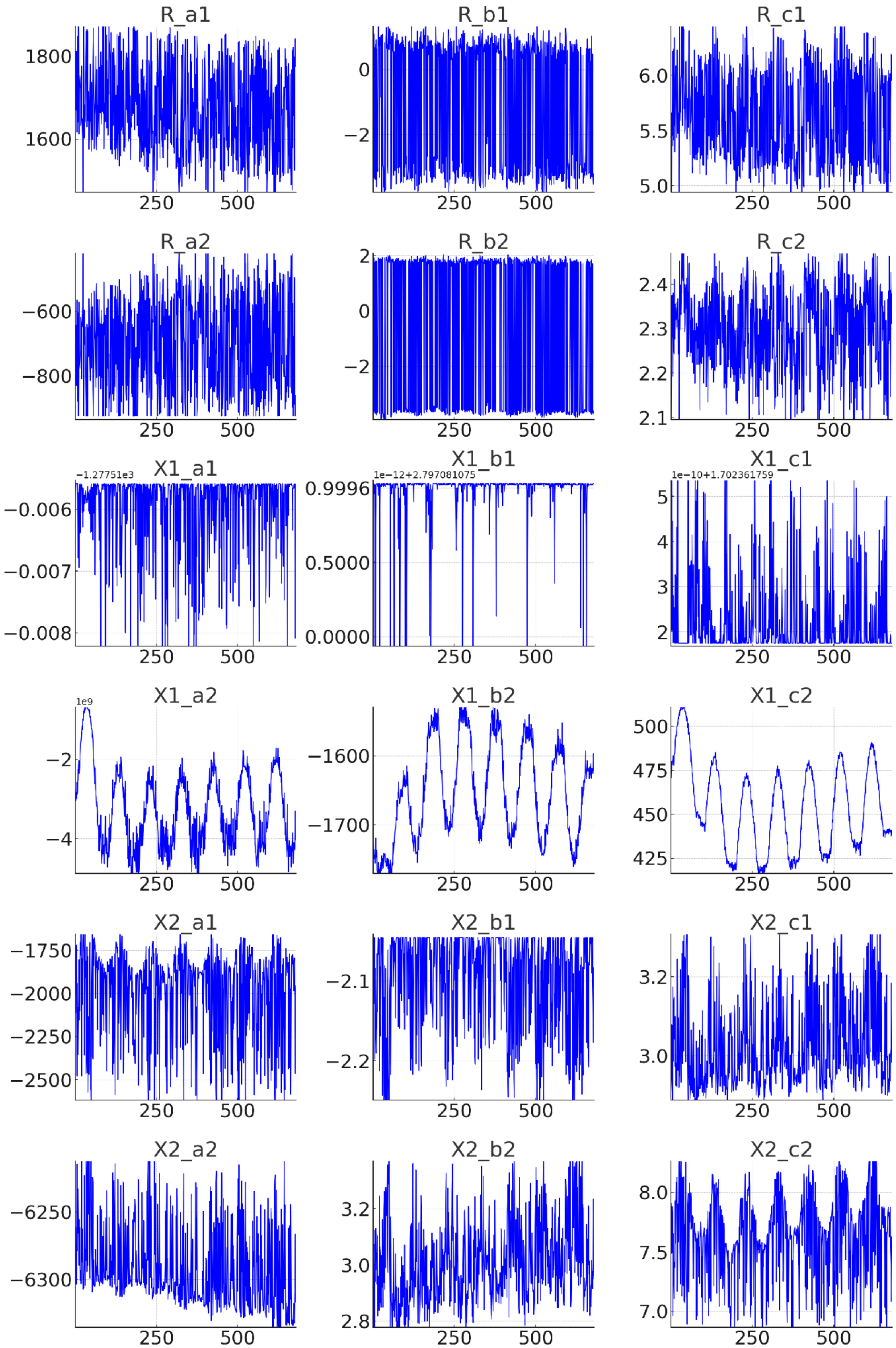

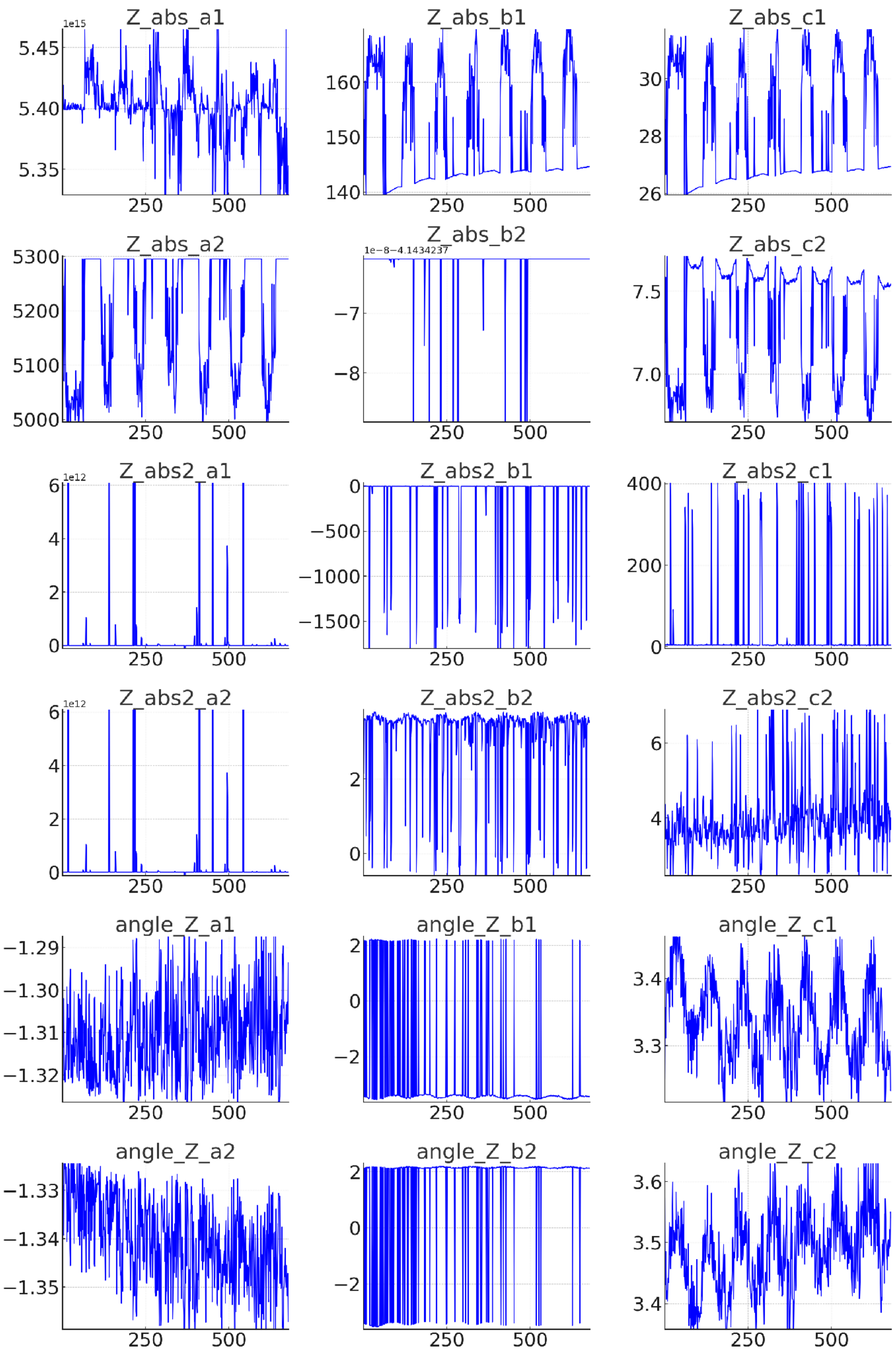
Z_abs_a1
Z_abs_b1
Z_abs_c1
Z_abs_a2
Z_abs_b2
Z_abs_c2
Z_abs2_a1
Z_abs2_b1
Z_abs2_c1
Z_abs2_a2
Z_abs2_b2
Z_abs2_c2
angle_Z_a1
angle_Z_b1
angle_Z_c1
angle_Z_a2
angle_Z_b2
angle_Z_c2

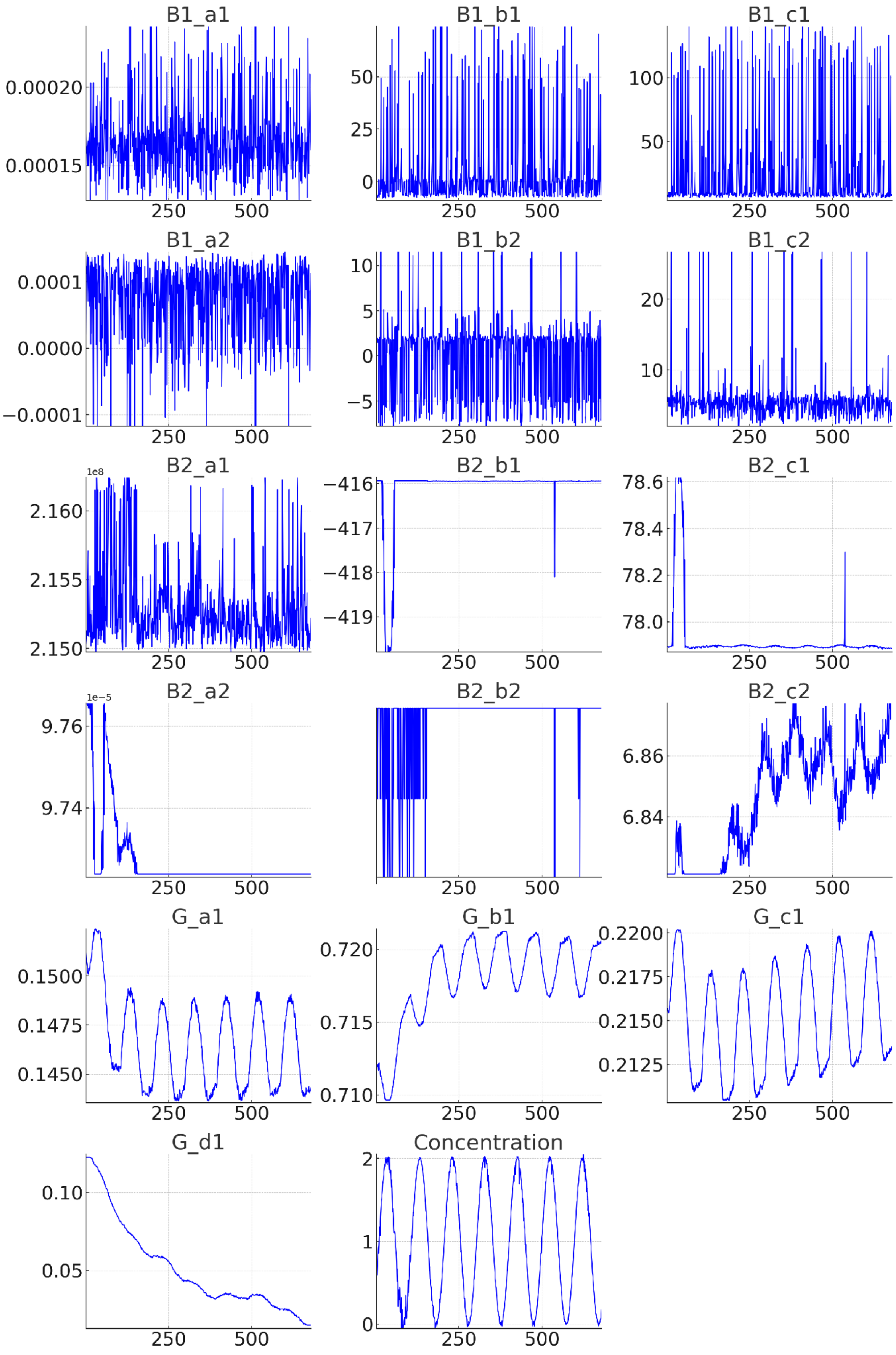
B1_a1
0.00020
0.00015
250
500
B1_b1
50
25
0
B1_c1
100
50
B1_a2
0.0001
0.0000
−0.0001
B1_b2
10
5
0
−5
B1_c2
20
10
B2_a1
1e8
2.160
2.155
2.150
B2_b1
−416
−417
−418
−419
B2_c1
78.6
78.4
78.2
78.0
B2_a2
1e−5
9.76
9.74
B2_b2
B2_c2
6.86
6.84
G_a1
0.1500
0.1475
0.1450
G_b1
0.720
0.715
0.710
G_c1
0.2200
0.2175
0.2150
0.2125
G_d1
0.10
0.05
Concentration
2
1
0

S.10. Best Performing Regression Models' Prediction Performances

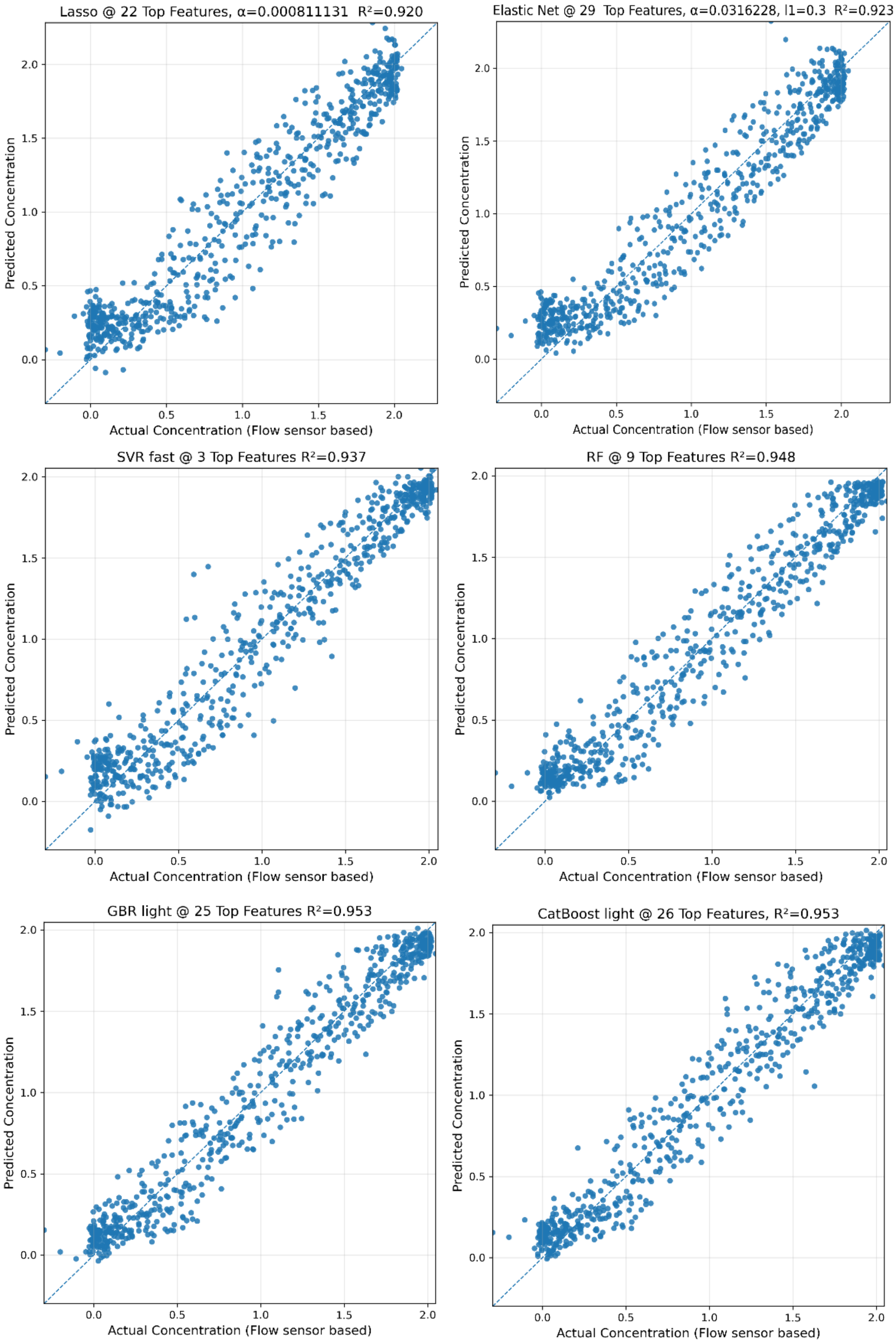

Supplement: Supplementary file 1 [file biosensors-16-00574-s001.zip › biosensors-4420825-supplementary.pdf]
